# Metabolite Profiling and Chemometric Study for the Discrimination Analyses of Geographic Origin of Perilla (*Perilla frutescens*) and Sesame (*Sesamum indicum*) Seeds

**DOI:** 10.3390/foods9080989

**Published:** 2020-07-24

**Authors:** Tae Jin Kim, Jeong Gon Park, Hyun Young Kim, Sun-Hwa Ha, Bumkyu Lee, Sang Un Park, Woo Duck Seo, Jae Kwang Kim

**Affiliations:** 1 Division of Life Sciences, College of Life Sciences and Bioengineering, Incheon National University, Incheon 22012, Korea; f91gd@inu.ac.kr (T.J.K.); parkjk1132@naver.com (J.G.P.); 2 Division of Crop Foundation, National Institute of Crop Science, Rural Development Administration, Wanju, Jeonbuk 55365, Korea; hkkim84@korea.kr; 3 Department of Genetic Engineering and Graduate School of Biotechnology, Kyung Hee University, Yongin 17104, Korea; sunhwa@khu.ac.kr; 4 Department of Environment Science & Biotechnology, Jeonju University, Jeonju 55069, Korea; leebk@jj.ac.kr; 5 Department of Crop Science, Chungnam National University, 99 Daehak-ro, Yuseong-gu, Daejeon 34134, Korea; supark@cnu.ac.kr

**Keywords:** perilla, sesame, geographic origin, metabolomics, multivariate analysis, metabolite profiling

## Abstract

Perilla and sesame are traditional sources of edible oils in Asian and African countries. In addition, perilla and sesame seeds are rich sources of health-promoting compounds, such as fatty acids, tocopherols, phytosterols and policosanols. Thus, developing a method to determine the geographic origin of these seeds is important for ensuring authenticity, safety and traceability and to prevent cheating. We aimed to develop a discriminatory predictive model for determining the geographic origin of perilla and sesame seeds using comprehensive metabolite profiling coupled with chemometrics. The orthogonal partial least squares-discriminant analysis models were well established with good validation values (*Q*^2^ = 0.761 to 0.799). Perilla and sesame seed samples used in this study showed a clear separation between Korea and China as geographic origins in our predictive models. We found that glycolic acid could be a potential biomarker for perilla seeds and proline and glycine for sesame seeds. Our findings provide a comprehensive quality assessment of perilla and sesame seeds. We believe that our models can be used for regional authentication of perilla and sesame seeds cultivated in diverse geographic regions.

## 1. Introduction

Perilla (*Perilla frutescens*) seed is a rich source of health-promoting compounds, such as tocopherols, phytosterols, policosanols and fatty acids, which have various bioactivities [1]. Tocopherols have an antioxidant effect and are known as vitamin E. Phytosterols show reduction of total cholesterols in the serum. They increase high-density lipoprotein cholesterol levels and reduce low-density lipoprotein cholesterol levels in the blood. Policosanols also have a serum lipid- and cholesterol-lowering effect and other beneficial effects, such as cytoprotection, antiaging, liver protection, antioxidant and anti-parkinsonian effects [2]. In addition, perilla seeds contain high levels of octacosanol (C28-ol) [1,2]. The fatty acid α-linolenic acid (C18:3n3) is found in high levels in perilla seeds, which is essential to human health; moreover, perilla seeds contain omega-3 fatty acid, which lowers inflammation and risk of cancer and cardiovascular and atopic diseases [3]. Sesame (*Sesamum indicum* L.) seeds also contain the abovementioned health-promoting compounds, and they are a good source of proteins rich in sulfur-containing amino acids [4,5]. Linoleic acid (C18:2n6), which is an essential fatty acid for humans, is the main fatty acid found in sesame seeds; in addition, oleic acid (C18:1n9) is the second most abundant fatty acid in sesame seeds [6]. In addition, γ-tocopherol is the main tocopherol in sesame seeds [6,7]. Sesame seeds reportedly contain high levels of phytosterols [5]. Although the composition and contents of various health beneficial compounds in the perilla and sesame seeds have been reported, to the best of our knowledge, a comprehensive comparative-analysis involving hydrophilic and lipophilic compounds has not been reported.

Metabolomics has been widely used to distinguish food products on the basis of differences in their chemical composition and metabolite contents [8,9]. Food metabolomics comprises analytical techniques and multivariate discriminant analysis (MVDA) techniques used for food substances. The analytical techniques usually used in food metabolomics are mass spectrometry (MS) coupled with separation techniques such as liquid chromatography (LC) and gas chromatography (GC) and nuclear magnetic resonance (NMR) [10]. For MVDA, the most commonly used methods are principal component analysis (PCA), partial least squares-discriminant analysis (PLS-DA) and orthogonal partial least squares discriminant analysis (OPLS-DA), which are useful tools for describing correlations and diagnosing differences among the studied samples and their metabolites. Therefore, food metabolomics strategies are suitable for analyzing food safety, authenticity, traceability and quality assessment and these strategies have been used to assess various foods and beverages, such as adzuki bean, olive oil, cabbage, wine, rice, coffee and tomato [11,12,13,14,15,16].

Perilla and sesame seeds are traditionally used as sources of edible oils in Korea, China, India and other Asian countries. Perilla is cultivated in Korea, China, Japan, India, Nepal and Thailand [17,18]. In Korea, the production of perilla seeds was average 40,448 tons per year over the last decade, and approximately 24,411 tons were imported per year [19]. Out of the imported perilla seeds, almost of 99% are Chinese perilla seeds [20]. Sesame is mainly produced in China, Myanmar, India and African countries such as Sudan, Nigeria and Tanzania. In Korea, the average production of sesame was 12,168 tons over the last decade, whereas approximately 76,812 tons were imported; the self-sufficiency rate in sesame production was 14% [19,20]. In particular, more than 90% of sesame seeds were imported from China (50%) and India (40%) [19]. The price of perilla and sesame seeds is influenced by their places of origin; therefore, identification of the geographic origin of these seeds is important [21]. Forging or mislabeling domestic seeds as imported seeds to gain economic benefits has increasingly become a crucial issue for both producers and consumers, and it affects food quality assurance and safety [22]. To prevent this problem, developing a precise and accurate method to identify the geographic origin of perilla and sesame seeds is needed. Recently, genomic and analytical approaches have been developed for such identification [4,6,15,23,24,25]. The genomics method is considerably accurate; however, it cannot determine the geographic origins of the same plant variety [14]. On the contrary, the analytical methods can accurately determine the different geographic origins of the same variety based on the differences in chemical composition. Previous studies have used multivariate analysis for discriminating between geographic origins of perilla and sesame seeds using genomics and analytical methods [4,22]. In the case of perilla, however, genomic methods have been reported to determine geographic origin, but analytical methods have not been developed [23].

We aimed to develop a method to discriminate the geographic origin of perilla and sesame seeds and to assess their nutritional quality. To discriminate the geographic origin, MVDA was performed with targeted metabolite profiling using gas chromatography-mass spectrometry (GC-MS). The hydrophilic and lipophilic metabolite profiling (including amino acids, organic acids, sugars, sugar alcohols, tocopherols, sterols, policosanols and fatty acids) of perilla and sesame seeds originated in the Korea and China was performed. Using this, a discrimination model was established for the determination of geographic origins of perilla and sesame seeds. This is the first attempt to construct a discrimination model for perilla seeds using metabolomics. Further, potential biomarkers for distinguishing the geographic origins of perilla and sesame seeds were proposed. A comprehensive food quality assessment was also performed. Our findings can offer reliable information about food authenticity and traceability of perilla and sesame seeds.

## 2. Materials and Methods

### 2.1. Sample and Chemicals

Korean perilla and sesame cultivars were grown at the National Institute of Crop Science, Rural Development Administration, Wanju-gun, Korea, during the 2018 growing season (June to November). Chinese perilla and sesame samples were procured from a local market in Xinzhou and JiangXia district (Wuhan city), China. The Chinese samples including perilla and sesame were from the recent harvests of November 2017 and 2016, respectively. Three biologic replicates were prepared for each sample. 5α-Cholestane, ribitol, pentadecanoic acid, fatty acid methyl ester (FAME) mixture, *N*-methyl-*N*-trimethylsilyl trifluoroacetamide (MSTFA) and pyridine were purchased from Sigma-Aldrich (St. Louis, Mo, USA). All other chemicals used in this study were reagent grade unless stated otherwise.

### 2.2. Extraction and Analysis of Hydrophilic Compounds

The extraction and analysis of hydrophilic compounds was performed as described previously [26]. A finely ground sample (10 mg) was mixed with 1 mL of a mixture of methanol, water and chloroform in the ratio 2.5:1:1 (*v/v*/v). Sixty microliters of ribitol (200 µg/mL) was added to the mixture as an internal standard (IS) and the mixture was incubated using a Thermomixer Comfort (model 5355, Eppendorf AG, Hamburg, Germany) at 37 °C for 30 min at a mixing frequency of 1200 rpm. The mixture was centrifuged at 16,000× *g* for 3 min. The upper layer (methanol/water phase) of 800 µL was pipetted into a fresh tube and mixed with 400 µL of water. The methanol/water fraction was centrifuged at 16,000× *g* for 3 min and 900 µL of the supernatant was collected into a fresh tube. The aliquots were evaporated for 2 h in a centrifugal concentrator (CC-105; TOMY, Tokyo, Japan) and freeze-dried for over 16 h. For derivatization, 80 µL of 2% methoxyamine hydrochloride (MOX) in pyridine (*w/v*) was added in freeze-dried samples and the mixture was incubated at 30 °C and 1200 rpm for 90 min using a Thermomixer Comfort (Eppendorf AG). Subsequently, 80 µL of MSTFA was added and the mixture was further incubated at 37 °C and 1200 rpm for 30 min. The hydrophilic compounds were separated on the GCMS-QP2010 Ultra system equipped with autosampler AOC-20i (Shimadzu, Kyoto, Japan) and a DB-5 column (30 m length, 0.25-mm diameter and 1.00 μm thickness). The temperatures for injection, interface and ion source were set at 280, 280 and 200 °C, respectively. The carrier gas was helium and the column flow rate was 1.1 mL/min. The temperature was held for 4 min at 100 °C, after which it was increased at a rate of 10 °C/min up to 320 °C and held for 11 min. The runtime was 4.00 to 37.00 min and the scan mode was used with a mass range of 45 to 600 *m/z*. The compounds were confirmed using standards and the Wiley9, NIST11 and OA TMS DB5 (Shimadzu) libraries (Table S1). For relative quantification, we used ribitol as an IS and the calculated the integrated peak area of all the analyte ratios relative to the peak area of the IS.

### 2.3. Extraction and Analysis of Lipophilic Compounds

Extraction and analysis of lipophilic compounds (policosanols, phytosterols, tocopherols and other terpenoids) was performed as described previously [27]. Finely ground samples weighing 10 mg were collected in 15-mL conical tubes, and 3 mL of ethanol containing 0.1% ascorbic acid (*w/v*) was added to the tubes. Fifty microliters of 5α-cholestane (10 µg/mL) was added to the mixture as an IS. Next, the samples were vortexed for 20 s and placed in a water bath at 85 °C for 5 min. Subsequently, 120 µL of potassium hydroxide (80%, *w/v*) was added for saponification, and the mixture was vortexed for 20 s. The mixture was returned to the water bath at 85 °C for 10 min. The samples were then cooled on ice for 5 min, and 1.5 mL each of deionized water and hexane was added to each sample and vortexed for 20 s. The mixture was centrifuged at 1200× *g* for 5 min at 4 °C and the upper layer was pipetted into afresh tube. In order to re-extract the remaining compounds, 1.5 mL of hexane was added again into the remaining pellets. The hexane fraction was collected in fresh tubes and evaporated under a stream of N_2_ gas in a centrifugal concentrator (TOMY). For the derivatization step, 30 µL of MSTFA and 30 µL of pyridine were added and incubated at 60 °C and 1200 rpm for 30 min using a Thermomixer Comfort (model 5355, Eppendorf AG, Hamburg, Germany). The GCMS-QP2010 Ultra system, equipped with the autosampler AOC-20i (Shimadzu), was installed with a Rtx-5MS column (30 m length, 0.25-mm-diameter and 0.25-µm-thickness) and used for the separation of lipophilic compounds. In total 1.0 µL of each sample was injected with split mode (10:1 ratio) and the injection temperature was set at 290 °C. Helium was used as a carrier gas and the column flow rate was 1.0 mL/min. The oven temperature was held for 2 min at 150 °C, increased at the rate of 15 °C/min up to 320 °C and finally held for 10 min. The chromatography runtime was 2.00–23.33 min. The MS interface and ion source temperatures were 280 and 230 °C, respectively. The Labsolutions GCMSsolution software version 4.20 (Shimadzu Kyoto, Japan) was used for the analysis of chromatograms and mass spectra. The calibration curve range of each lipophilic compound was 0.025–5.00 µg, and a fixed concentration (0.50 µg each) of the internal standard was used. Qualitative and quantitative analyses were conducted using standards (Table S2).

Extraction of fatty acids was performed according to a method described previously, but with slight modifications [28,29]. Briefly, 10-mg of sample was mixed with 2.5 mL of chloroform/methanol (2:1, *v/v*) and 10 μL of pentadecanoic acid (100 μg/mL) as an IS. The mixture was sonicated for 15 min. Next, 2.5 mL of 0.58% (*w/v*) sodium chloride (NaCl) in water was added to separate the extract into two phases (methanol-water and chloroform) and to remove proteinaceous matter from the chloroform fraction. The mixture was briefly vortexed and then centrifuged at 13,000× *g* for 5 min at 4 °C. Thereafter, the chloroform phase (bottom layer) was pipetted into a new tube and evaporated using a centrifugal concentrator (TOMY). Toluene (100 μL), 5 M sodium hydroxide (NaOH, 20 μL) and methanol (180 μL) were added to the dried sample, and the tube was incubated at 85 °C for 5 min. Next, 300 μL of 14% (*w/v*) boron trifluoride (BF_3_) in methanol was added for methylation, and the reaction was performed at 85 °C for 5 min. Afterward, 800 μL of pentane and 400 μL of distilled water were added to the tube, and the tube was centrifuged at 750× *g* for 15 min at 4 °C. The supernatant was collected into a new 2-mL tube and concentrated using the centrifugal concentrator. The concentrated sample was finally dissolved in 300 μL of hexane, filtered through a 0.5-μm syringe filter and analyzed by gas chromatography–quadrupole mass spectrometry (GC-qMS) (Shimadzu). The methylated fatty acids (1 μL) were separated in a DB-5 column (30 m × 0.25 mm × 1.00 μm; Agilent, Palo Alto, CA, USA) using a GCMS-QP2010 Ultra system with autosampler AOC-20i (Shimadzu). Injection volume of the samples was 1.0 µL and split mode was set at 10:1 ratio. Injection, ion source and interface temperatures were set at 280 °C, 200 °C and 280 °C, respectively. The column temperature conditions were as follows. The initial temperature was maintained at 40 °C for 2 min and raised to 320 °C at a rate of 6 °C/min. Helium was used as a carrier gas at a flow rate of 1.42 mL/min. Runtime was 2.86 to 49.00 min and scan mode was used with a mass range of 45 to 500 *m/z*. Qualitative and quantitative analyses of fatty acids were conducted using standards and a FAME Mix (C8–C24) (Table S3).

### 2.4. Statistical Analysis

All analyses were performed no fewer than three times. Data obtained from GC-qMS were analyzed using PCA and OPLS-DA (SIMCA-P version 13.0; Umetrics, Umea, Sweden) to discriminate the geographic origin of perilla and sesame seeds. To determine the optimal OPLS-DA model, all the data were normalized with unit variance (UV)-scaling and pareto-scaling. PCA and OPLS–DA were based on the calculated eigenvectors and eigenvalues. The external validation test, permutation test and analysis of variance of the cross-validated residuals (CV-ANOVA) were conducted using SIMCA-P version 13.0 (Umetrics). The receiver operating characteristic (ROC) analysis and student’s *t*-test were performed using MetaboAnalyst 4.0 (https://www.metaboanalyst.ca).

## 3. Results

### 3.1. Metabolite Profiling of Perilla and Sesame Seeds

To discriminate the geographic origin of perilla and sesame seeds, we analyzed hydrophilic and lipophilic compounds using GC-qMS. We detected 35 hydrophilic compounds in 19 samples of perilla seeds and 31 hydrophilic compounds in 25 samples of sesame seeds (Tables S4 and S5). The lipophilic compounds, such as fatty acids, sterols, policosanols and tocopherols, were detected and quantified in all seed samples (Tables S6–S11). In total, 28 lipophilic compounds, including 11 fatty acids, 9 policosanols, 3 tocopherols, 3 sterols and 2 amyrins, were identified in perilla seeds (Tables S6, S8 and S10). In addition, 23 lipophilic compounds, including 10 fatty acids, 9 policosanols, 1 tocopherol and 3 sterols were detected in sesame seeds (Tables S7, S9 and S11). Unlike perilla seeds, α- and β-tocopherols, α- and β-amyrins and C18:3n3 were not detected in sesame seeds.

### 3.2. PCA and OPLS-DA for Geographic Discrimination of Perilla and Sesame Seeds

To discriminate the geographic origins of perilla and sesame seeds, the metabolite profiling data were processed using multivariate statistical analysis (PCA and OPLS-DA), which is an important tool for identifying the features of samples in complex data matrices. PCA uses an orthogonal linear transformation to transform the original data into a new set of variables, the principal component (PC). The scores and loading of PCs are represented in a bi-dimensional plot, which can formulate a dataset pattern from the raw data. The data were normalized with UV-scaling. In the PCA score plots, the two seeds did not show any variance according to geographic origins (Figures S1 and S2).

To improve the geographic discrimination of perilla and sesame seeds, we used OPLS-DA to determine the differences in metabolites arising due to differences in the geographic origin. OPLS-DA is a supervised classification method that features (X variables: metabolites) divides into two parts to separate the systematic variation: one that models the correlation between X and Y (prediction) and another that models the orthogonal components [30]. Thus, OPLS-DA has maximum separation by geographic origins based on their metabolites. The geographic origins (Y-variables) were set to 0 for Korea and 1 for China. Internal validation method was used to validate the model. The quality of the predictive model was measured by *R*^2^ and *Q*^2^ values of the validation results. The *R*^2^ value indicates how much the proportion of variation in the data is explained by the model and the goodness of fit. The *Q*^2^ value indicates how much proportion of variation in the data is predictable by the model and the goodness of prediction. The parameters *R*^2^ and *Q*^2^ were calculated minimum zero to maximum one; the *R^2^* value closer to 1 indicates a good value, *Q*^2^ > 0.5 is regarded as a good prediction model and *Q*^2^ > 0.9 is regarded as excellent prediction model. To develop a better discrimination model, the data were normalized by UV and pareto scaling. The optimal OPLS-DA model was established using UV-scaling, which showed higher *R*^2^Y (perilla; 0.822, sesame; 0.844) and *Q*^2^ (perilla; 0.761, sesame; 0.799) values than pareto-scaling (*R*^2^Y: perilla; 0.575, sesame; 0.744/ *Q*^2^: perilla; 0.480, sesame; 0.715) (Table 1). The OPLS-DA models of both perilla and sesame seeds showed the *Q*^2^ values to be above 0.5, indicating a good prediction model.

**Figure 1:**
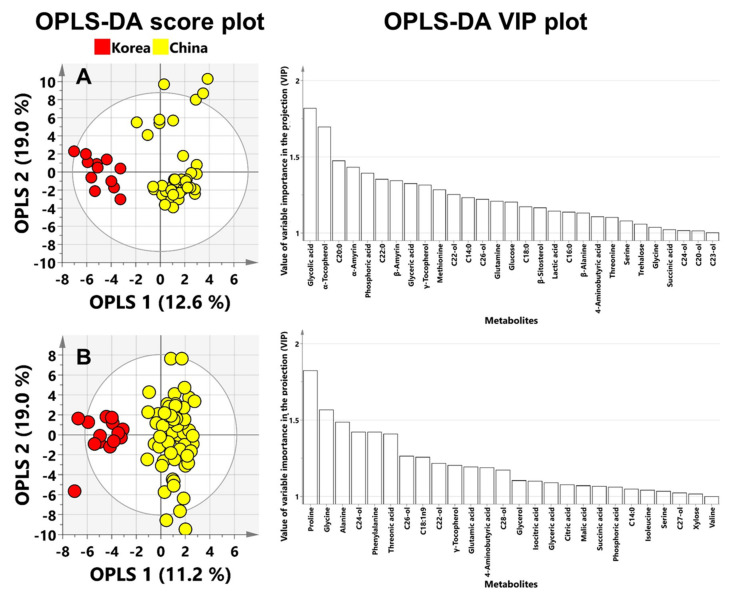
OPLS–DA score plots and VIP (variable importance in the projection) plots of (**A**) perilla and (**B**) sesame seeds from Korea and China. C20-ol—eicosanol; C21-ol—heneicosanol; C22-ol—docosanol; C23-ol—tricosanol; C24-ol—tetracosanol; C26-ol—hexacosanol; C27-ol—heptacosanol; C28-ol—octacosanol; C30-ol—triacontanol; C12:0—lauric acid; C14:0—myristic acid; C16:1n7—palmitoleic acid; C16:0—palmitic acid; C18:2n6—linoleic acid; C18:3n3—α-linolenic acid; C18:1n9—oleic acid; C18:0—stearic acid; C20:0—arachidic acid; C22:0—behenic acid; C24:0—lignoceric acid.

The OPLS-DA analysis was performed with UV-scaling data. The OPLS score plot of perilla seeds showed good separation on the basis of geographic origins (Korea and China) (Figure 1A). To identify the potential biomarkers for the geographic discrimination of perilla seeds, variable importance in projection (VIP) plots were used to explain the contribution of metabolites to the prediction models wherein VIP values greater than 1.00 indicate the significant influence on the model. In total, 29 metabolites had greater than 1.00 VIP values (Table S12). Glycolic acid, α-tocopherol and C20:0 were top-ranked metabolites in the VIP plots. The OPLS score plot of sesame seeds also showed good separation by region (Korea and China) (Figure 1B). In total, 26 metabolites showed a VIP cut off value of over 1.00 (Table S13). Proline, glycine and alanine were top-ranked in VIP plots.

**Figure 2:**
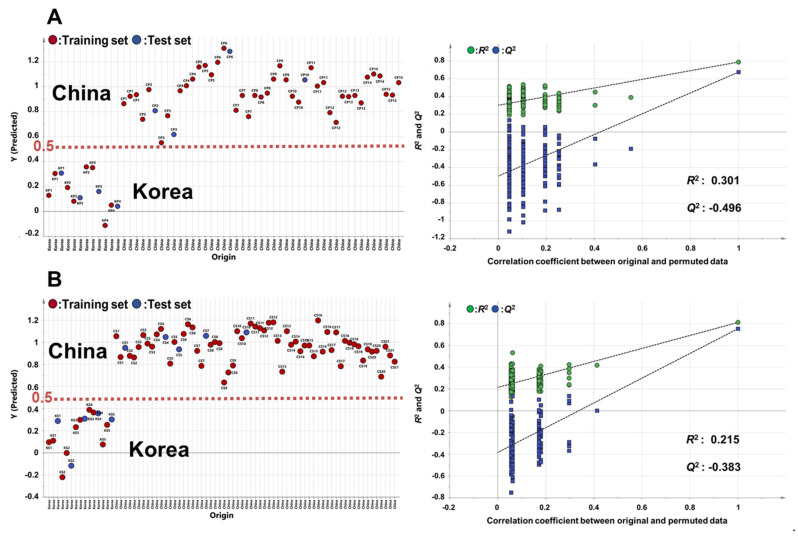
External validation test and permutation test by OPLS-DA for discriminating the geographic origin of (**A**) perilla and (**B**) sesame seeds from Korea and China. The number of permutations for the permutation test was 200. (A: *R*^2^X = 0.298, *R*^2^Y = 0.788, *Q*^2^ = 0.674, RMSEP = 0.229; B: *R*^2^X = 0.320, *R*^2^Y = 0.812, *Q*^2^ = 0.754, RMSEP = 0.208).

The established OPLS-DA model for the discrimination of perilla and sesame seeds on the basis of geographic origin was subjected to an external validation test to determine its accuracy. In the case of perilla seeds, 57 samples were divided into 49 training samples and 8 test samples. The Y-variables were set to 0 for Korea and 1 for China. The OPLS projection model was established using 49 training samples, and then the 8 test samples were projected on the established OPLS projection model. The results of external validation test showed good discrimination of geographic origin of perilla seeds in the OPLS prediction model with *R*^2^X = 0.298, *R*^2^Y = 0.788 and *Q*^2^ = 0.674. In addition, this OPLS model showed a root mean square error of prediction (RMSEP) = 0.229, which indicates the accuracy of prediction. The RMSEP value, being close to zero, indicated a good value. Furthermore, perilla seeds cultivated in Korea and China did not fall on the borderline of 0.5, which was a threshold level in the external validation test. Additionally, a permutation test and CV-ANOVA were conducted to test the risk of over-fitting the OPLS model. The permutation test was performed with 200 permuted models, which was constructed using randomized Y-variables. The reference distribution of the *Q*^2^ value for random data from permuted models was compared with the *Q^2^* value of the real (unpermuted) OPLS model. When the *Q^2^* value from the permuted model is smaller than the *Q^2^* value of the original OPLS model, the model is considered as a predictable model. The results of the permutation test showed the *Q*^2^ value of −0.496, which was lower than the *Q*^2^ value of the original OPLS model (Figure 2A). The CV-ANOVA test was performed to testify the validity of the model. When the *p*-value was smaller than 0.05, the model was regarded as a validated model. The *p*-value of perilla seeds from the CV-ANOVA test was 3.05 · 10^−10^.

To perform the external validation test for the OPLS-DA model of sesame seeds, the 78 samples were divided into 68 training samples and 10 test samples. The 68 training samples were used for the construction of the OPLS prediction model, and the 10 test samples were projected on the OPLS model. The external validation test results displayed good separation of sesame seeds samples on the basis of geographic origin in the OPLS projection model, which showed validation values with *R*^2^X = 0.320, *R*^2^Y = 0.812, *Q*^2^ = 0.754 and RMSEP = 0.208. The results of the permutation test for the OPLS predictive model for sesame seeds showed the *Q*^2^ value of −0.383, which was smaller than the *Q*^2^ value of the real OPLS model. The CV-ANOVA test results of sesame seeds showed the *p*-value of 1.61 · 10^−18^. Therefore, the OPLS-DA model for geographic discrimination of both of perilla and sesame seeds were successfully established and validated.

### 3.3. Potential Biomarkers for the Discrimination of Perilla and Sesame Seeds Based on Their Geographic Origins

The OPLS-biplot displayed a combination of observations (samples), X-variables (metabolites) and Y-variables (geographic origin) in a bi-dimensional space. This could easily explain the correlation of variables and the clustering of samples. The three ellipses—inner (0.50), middle (0.75) and outer (1.00)—indicate that the explained variances are 50%, 75% and 100%, respectively. If the variables are located close to the observations, the sample group has high levels of metabolites, whereas if they are opposite, the levels of metabolites are low. If the variables are closer to the outer circle (1.00) of the OPLS-biplot, the metabolites have more significantly contributed to the model.

**Figure 3:**
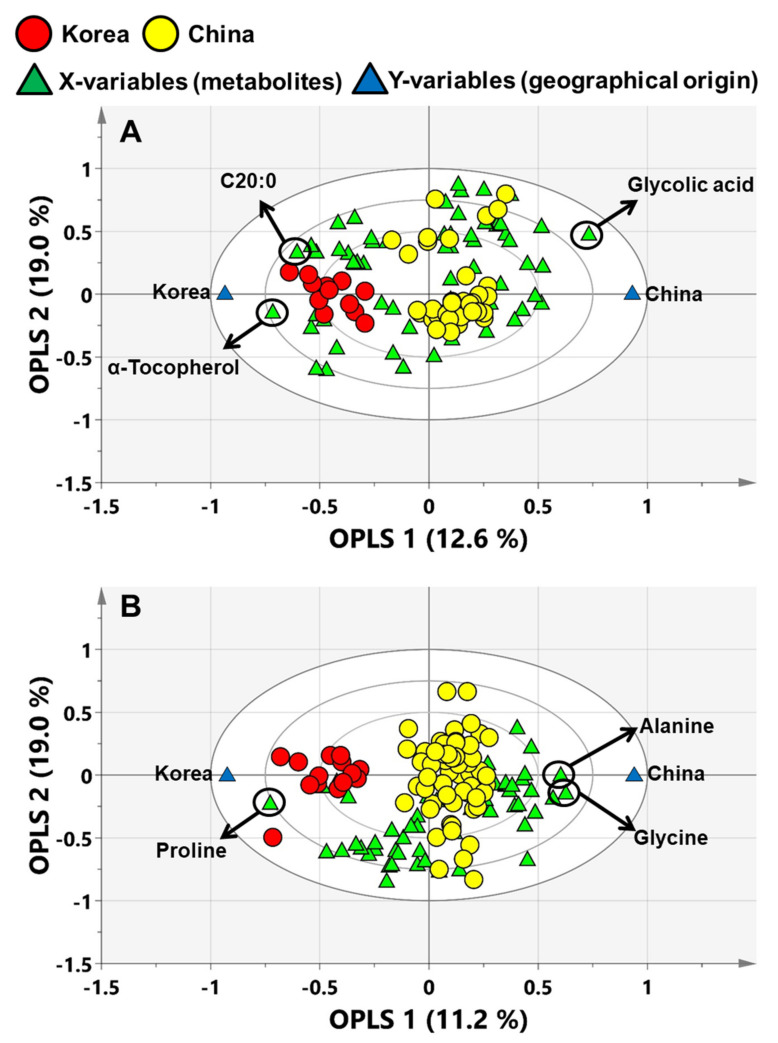
The OPLS-biplot for discriminating the geographic origin of (**A**) perilla and (**B**) sesame seeds using metabolite profiling data. The OPLS-biplot showed correlation of all metabolites (X-variables), sample clusters (observations) and geographic origins (Y-variables). C20:0; arachidic acid.

**Figure 4:**
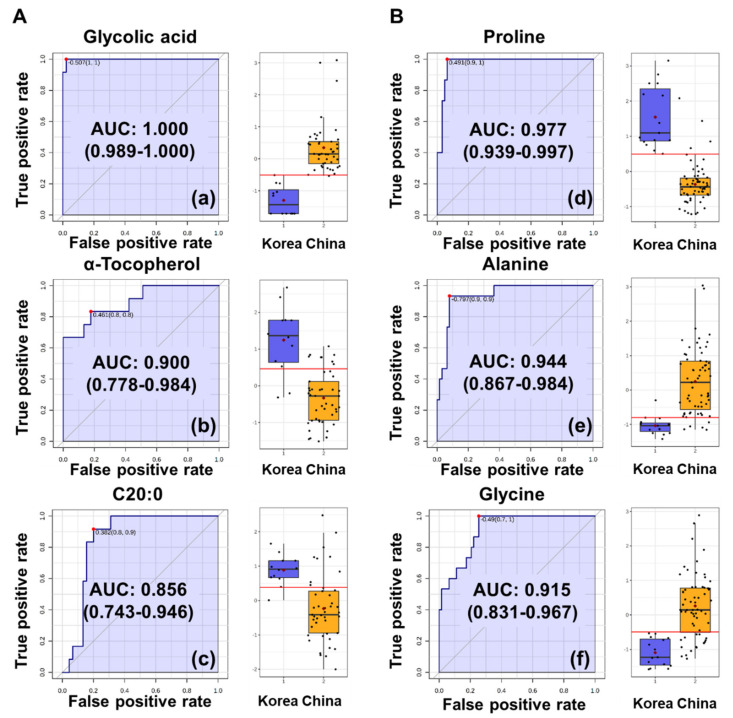
Receiver operating characteristic (ROC) curves for discriminating the geographic origins of (**A**) perilla and (**B**) sesame seeds using metabolite profiling data. ROC curves for (a) glycolic acid, (b) α-tocopherol and (c) C20:0 (arachidic acid) on discriminating (**A**) perilla seeds from Korea and China. ROC curves for (d) proline, (e) alanine and (f) glycine on discriminating (**B**) sesame seeds from Korea and China.

In the OPLS-biplot of perilla seeds, glycolic acid, α-tocopherol and C20:0 were significant contributors, which were notably positioned the closest to the outer (1.00) circle and Y-variables (Figure 3A). In particular, only glycolic acid was located within middle (0.75) and outer (1.00) circles among these metabolites. In addition, these metabolites had top-ranked VIP values (glycolic acid, 1.82; α-tocopherol, 1.70; and C20:0, 1.48) in VIP plot. Therefore, to evaluate the predictive performance of these metabolites as potential biomarkers, ROC analysis was conducted. When the area under curve (AUC) values, which were a result of the ROC analysis, are to be closer to 1.00, the outcome is desirable [4]. Glycolic acid showed the AUC value of 1.000, indicating the excellent accuracy of discriminating Korean and Chinese perilla seeds (Figure 4A). In addition, α-tocopherol (AUC: 0.900) and C20:0 (AUC: 0.856) showed good accuracy to be considered as potential biomarkers. Therefore, glycolic acid was proposed as a potential biomarker for Chinese perilla seeds.

As shown in Figure 4B, proline, glycine and alanine, which were top-ranked (proline, 1.82; glycine, 1.57; and alanine, 1.49) in the VIP plot of sesame seeds, were located the closest to the outer circle and Y-variables. These metabolites showed AUC values in the range of 0.915–0.944, indicating their excellent accuracy as potential biomarkers for discriminating Korean and Chinese sesame seeds. Thus, proline, glycine and alanine were proposed as potential biomarkers for discriminating sesame seeds on the basis of geographic origin.

## 4. Discussion

The quality of perilla and sesame seeds and oils based on various health-related compounds such as fatty acids, tocopherols and sterols has been assessed previously [1,5]. However, to the best of our knowledge, a comprehensive metabolite profiling, which combines primary and secondary metabolites, has not been reported for perilla and sesame seeds. Therefore, we analyzed the primary metabolites and health-promoting compounds, which are abundantly found in perilla and sesame seeds, using GC-qMS. Perilla and sesame seeds are important oil crops, and they contain high levels of lipophilic compounds. In our analysis, perilla seeds showed high levels of α-linolenic acid (C18:3n3) and linoleic acid (C18:2n6), which are essential omega-3 and -6 fatty acids, respectively (Tables S10 and S11). On the contrary, α-linolenic acid (C18:3n3) was not detected in sesame seeds. However, linoleic acid (C18:2n6) and oleic acid (C18:1n9) were detected in higher levels in sesame seeds than in perilla seeds. Among tocopherols, γ-tocopherol was found in the highest amount in both perilla and sesame seeds; however, α- and β-tocopherols were not detected in sesame seeds. Phytosterols were found in high amounts in perilla and sesame seeds (Tables S8 and S9). The levels of phytosterols in sesame seeds were approximately three times higher than those in perilla seeds. The above results were consistent with those of the previous studies [1]. Perilla seeds showed high levels of policosanols (Table S6). In particular, C28-ol was found in the highest level among policosanols in perilla seeds. However, sesame seeds showed low levels of policosanols (Table S7). These results agreed with those of the previous studies, which showed that perilla seeds and oils contain the highest levels of policosanols among other oil crops, while sesame seeds and oils contain negligible amounts of policosanols [31,32]. The hydrophilic metabolites, such as amino acids, organic acids and sugars, were detected in both perilla and sesame seeds (Tables S4 and S5). Almost all amino acids were found at higher levels in sesame seeds than in perilla seeds, except methionine and β-alanine. Sesame seeds are known as a good source of proteins rich in high sulfur-containing amino acids [4,5]. Therefore, sesame seeds may be consumed methionine for generating protein, which including high sulfur-containing amino acids. For the synthesis of high amount methionine, aspartic acid metabolism is activated. As a result, aspartic acid levels were higher in sesame seeds than in perilla seeds. In addition, sesame seeds have high levels of phenylalanine. Sesame seeds are also known to contain high amounts of lignans such as sesamin, sesamolin and sesamol [6,7]. Therefore, sesame seeds may have an activated phenylpropanoid pathway for the synthesis of lignans, resulting in the upregulated levels of phenylalanine.

To compare the compositional differences in seeds according to their origins, student’s *t*-test was performed with metabolite profile data of perilla and sesame seeds. The *t*-test results of perilla seeds showed that 22 metabolites were considered statistically significant (0.05 ≥ *p*-value) between Korean and Chinese perilla seeds. In addition, these metabolites were shown to have compositional differences with geographic origins of perilla seeds. In the OPLS-DA loading plots of perilla seeds, the Korean perilla seeds had higher amounts of five terpenoids (α-, γ-tocopherols, β-sitosterol and α-, β-amyrin), five fatty acids (C14:0, C16:0, C18:0, C20:0 and C22:0) and methionine than Chinese seeds (Figure S3B). On the other hand, four policosanols (C20-ol, C22-ol, C24-ol and C26-ol), five organic acids (glycolic acid, phosphoric acid, nicotinic acid, lactic acid, glyceric acid), 4-aminobutyric acid and sucrose were shown to be present in higher levels in Chinese perilla seeds. In the case of sesame seeds, 25 metabolites were considered statistically significant between Korean and Chinese seeds. In the OPLS-DA loading plots of sesame seeds, three fatty acids (C14:0, C18:1n-9 and C24:0), four organic acids (citric acid, isocitric acid, malic acid and threonic acid), threonine and C22-ol were higher in concentration in Korean sesame seeds than in Chinese sesame seeds (Figure S4B). Whereas, the Chinese sesame seeds contained higher amounts of four amino acids (glycine, alanine, phenylalanine and 4-aminobutyric acid), two organic acids (succinic acid and glyceric acid), four policosanols (C24-ol, C28-ol, C26-ol and C30-ol), γ-tocopherol, glycerol, phosphoric acid, inositol and fructose than the Korean sesame seeds.

We determined and predicted the geographic origins of perilla and sesame seeds cultivated in China and Korea using OPLS-DA (Figure 1). The score plot of OPLS-DA showed good separation of both perilla and sesame seeds using appropriate data pretreatment. The optimal data preprocessing method for the OPLS-DA model was the UV-scaling method with the highest *Q*^2^ and *R*^2^Y values in both of perilla and sesame seeds (Table 1). The selection of normalization methods is particularly important to reduce the unwanted instrumental errors of peak intensity measurements for relevant biologic differences. Thus, data normalization and scaling strategies should be chosen in such a way that the model shows optimal predictive ability of MVDA and retains meaningful biologic information [33].

The OPLS-biplots and VIP plots were generated to identify the biomarkers for discriminating perilla and sesame seeds on the basis of their geographic origins. Glycolic acid, α-tocopherol and C20:0 were identified as potential biomarkers for perilla seeds discrimination. Furthermore, proline, alanine and glycine were found to be potential biomarkers for sesame seeds discrimination. These potential biomarkers were further validated using ROC curve analysis. All AUC values of potential biomarkers were higher than 0.85, indicating that these metabolites significantly contribute to discriminating the seeds on the basis of their geographic origins. Kim et al. have reported that the VIP values of proline and glycine derived from the OPLS-DA model for discriminating the geographic origin of sesame seeds were higher than 1.0, indicating that these metabolites can be potential biomarkers for determining the regional origins of sesame seeds [4]. Thus, our results were consistent with those of a previous study. Glycolic acid is generated during photorespiration. Under low atmospheric CO_2_ condition, C3 photosynthetic metabolism fixes the competing substrate O_2_ instead of CO_2_. The oxygen fixation generates one molecule of 3-phosphoglycerate (3-PGA) and one molecule of 2-phosphoglycolate (2-PG) instead of two molecules of 3-PGA. Glycolic acid is generated from the dephosphorylation of 2-PG, and it can inhibit the rate of photosynthesis in the chloroplast. As a result, photorespiration under current atmospheric CO_2_ concentrations reduces the efficiency of C3 photosynthesis by ~15% to 50%, depending upon the temperature in the growing season at that particular geographic location [34]. Therefore, this study suggests that glycolic acid could be a potential biomarker for geographic discrimination of perilla seeds and proline and glycine could be the same for sesame seeds.

Outlier detection is an important issue in chemometrics analysis. The outliers are observations that are extreme or that do not fit the PCA model. Furthermore, outliers can be both serious and interesting observations in the data. To discover the outliers in the PCA model, we used the Hotelling’s *T*^2^. The Hotelling’s *T*^2^ is a multivariate generalization of student’s *t*-test and provides a check for observations adhering to multivariate normality. In the PCA score plots, the ellipse of Hotelling’s *T*^2^ indicates 95% confidence. When observations fall outside the confidence ellipse, they are termed as strong outliers. Observations suggested as outliers were removed from the entire data set. This process was repeated until no outliers were displayed on the PCA score plot. Figures S5 and S6 show the outlier removal process. A total of 11 samples were identified as outliers, and 46 samples remained in the data set of perilla seeds. In the OPLS-DA score plot of perilla seeds (Figure 1), Chinese perilla seeds were more dispersed than Korean perilla seeds because the outliers were clustered in the upper right of the score plot (Figure S3A). In addition, the data set of sesame seeds retained 69 samples and eliminated 9 samples. These pretreated data sets of perilla and sesame seeds were subjected to OPLS-DA. Figure S7 shows OPLS-DA scores and VIP plots of the outlier removal data sets. The OPLS-DA model was established using UV-scaling, which showed higher *R*^2^Y (perilla; 0.928, sesame; 0.876) and *Q*^2^ (perilla; 0.874, sesame; 0.842) values than the original data set *R*^2^Y (perilla; 0.822, sesame; 0.844) and *Q*^2^ (perilla; 0.761, sesame; 0.799) values. The OPLS-DA score plots for the outlier removal data sets showed good separation of both perilla and sesame seeds. In particular, the OPLS-DA score plots of the outlier removal data set of perilla seeds showed clearer clustering of the Chinese samples than that of the original data set. Furthermore, the VIP plots of the outlier removal data sets of perilla and sesame seeds showed results that were almost same as those of the original data sets. Although the number of samples was reduced by more than 10% due to the outlier removal, the potential biomarker candidates were the same as those from the original data sets. These results demonstrated that the established OPLS-DA discrimination models for perilla and sesame seeds were reliable predictive models.

In conclusion, we performed comprehensive metabolite profiling, which included primary metabolites and health-promoting secondary metabolites, for perilla and sesame seeds cultivated in Korea and China. In addition, we established the OPLS-DA discriminative model for perilla and sesame seeds and validated it with good test results. The OPLS-DA results showed a clear separation of perilla and sesame seeds sourced from Korea and China on the basis of their geographic origins. The OPLS-biplot and VIP plot showed that glycolic acid was a notable metabolite for discrimination of perilla seeds based on geographic origin; therefore, we propose it as a potential biomarker for such discrimination. Furthermore, proline and glycine most significantly contributed for determining the geographic origins of sesame seed, and thus, they could be potential biomarkers for discrimination of sesame seeds based on the geographic origin. This study provides a reliable discriminatory predictive model to determine the geographic origins of perilla and sesame seeds cultivated in Korea and China. In addition, to the best of our knowledge, this is the first attempt to construct a discrimination model for perilla seeds using metabolomics. We believe that this model will be helpful in dealing with issues of selling domestic perilla and sesame seeds in the name of imported ones. In this study, the number of samples and their source countries was limited. A future work should involve a larger sample size from more cultivated regions in various countries and evaluate the predictive ability of this model.

## Figures and Tables

**Table 1:** Model validation results from orthogonal partial least squares discriminant analysis (OPLS–DA) with various scaling methods for discriminating the geographic origin of perilla and sesame seeds.

Sample	X Variables Number	Scaling Method	*R^2^*X	*R^2^*Y	*Q^2^*
Perilla	57	UV	0.316	0.822	0.761
		Par	0.473	0.575	0.480
Sesame	78	UV	0.303	0.844	0.799
		Par	0.526	0.744	0.715

UV—unit variance; Par—pareto.

## Supplementary Materials

The following are available online at https://www.mdpi.com/2304-8158/9/8/989/s1, Figure S1: PCA score (A) and loading (B) plots of perilla (*Perilla frutescens*) seeds from Korea and China, Figure S2: PCA score (A) and loading (B) plots of sesame (*Sesamum indicum*) seeds from Korea and China, Figure S3: OPLS-DA score (A) and loading (B) plots of perilla (*Perilla frutescens*) seeds from Korea and China, Figure S4: OPLS-DA score (A) and loading (B) plots of sesame (*Sesamum indicum*) seeds from Korea and China, Figure S5: PCA score plots and Hotelling’s *T*^2^ range column plots of perilla (*Perilla frutescens*) seeds from Korea and China for outlier removal process, Figure S6: PCA score plots and Hotelling’s *T*^2^ range column plots of sesame (*Sesamum indicum*) seeds from Korea and China for outlier removal process, Figure S7: OPLS–DA score plots and VIP (variable importance in the projection) plots of perilla (A) and sesame (B) seeds from Korea and China outlier removal data sets, Table S1: Relative retention times (RRT) and mass spectral data of hydrophilic compounds as trimethylsilyl derivatives, Table S2: Relative retention times (RRT) and mass spectral data of lipophilic compounds as trimethylsilyl derivatives, Table S3: Relative retention times (RRT) and concentration of fatty acid methyl esters (FAME) mixture and fatty acids, Table S4: Composition and content (ratio/g) of hydrophilic compounds in perilla (*Perilla frutescens*) cultivars, Table S5: Composition and content (ratio/g) of hydrophilic compounds in sesame (*Sesamum indicum*) cultivars, Table S6: Composition and content (µg/g) of policosanol compounds in perilla (*Perilla frutescens*) cultivars, Table S7: Composition and content (µg/g) of policosanol compounds in sesame (*Sesamum indicum*) cultivars, Table S8: Composition and content (µg/g) of sterol and terpenoid compounds in perilla (*Perilla frutescens*) cultivars, Table S9: Composition and content (µg/g) of sterol and terpenoid compounds in sesame (*Sesamum indicum*) cultivars, Table S10: Composition and content (mg/g) of fatty acids in perilla (*Perilla frutescens*) cultivars, Table S11: Composition and content (mg/g) of fatty acids in sesame (*Sesamum indicum*) cultivars, Table S12: OPLS-DA loading plots and VIP values of variables of perilla (*Perilla frutescens*) cultivars, Table S13: OPLS-DA loading plots and VIP values of variables of sesame (*Sesamum indicum*) cultivars.
