# Supplementary material for: Metabolite Profiling and Chemometric Study for the Discrimination Analyses of Geographic Origin of Perilla (Perilla frutescens) and Sesame (Sesamum indicum) Seeds
Source: Foods. 2020 Jul 24;9(8):989. doi: 10.3390/foods9080989 (PMC7466206; doi:10.3390/foods9080989)
Supplement: Supplementary file 1 [file foods-09-00989-s001.zip › (Proof)PS_supplementary Tables_revision_final_ver.docx]

Supplementary tables

Metabolite Profiling and Chemometric Study for the Discrimination Analyses of Geographic Origin of Perilla (*Perilla frutescens*) and Sesame (*Sesamum indicum*) Seeds

Tae Jin Kim ^1, †^, Jeong Gon Park ^1, †^, Hyun Young Kim ^2^, Sun-Hwa Ha ^3^, Bumkyu Lee ^4^, Sang Un Park ^5^, Woo Duck Seo ^2,^* and Jae Kwang Kim ^1,^*

^1^ Division of Life Sciences, College of Life Sciences and Bioengineering, Incheon National University., Incheon, 22012, Republic of Korea; [f91gd@inu.ac.kr](mailto:f91gd@inu.ac.kr) (T.J.K.); [parkjk1132@naver.com](mailto:parkjk1132@naver.com) (J.G.P.)

^2^ Division of Crop Foundation, National Institute of Crop Science, Rural Development Administration, Wanju, Jeonbuk, 55365, Republic of Korea; [hkkim84@korea.kr](mailto:hkkim84@korea.kr) (H.Y.K.)

^3^ Department of Genetic Engineering and Graduate School of Biotechnology, Kyung Hee University, Yongin 17104, Republic of Korea; [sunhwa@khu.ac.kr](mailto:sunhwa@khu.ac.kr) (S.-H.H.)

^4^  Department of Environment Science & Biotechnology, Jeonju University, Jeonju 55069, Republic of Korea; [leebk@jj.ac.kr](mailto:leebk@jj.ac.kr) (B.L.)

^5^  Department of Crop Science, Chungnam National University, 99 Daehak-ro, Yuseong-gu, Daejeon 34134, Republic of Korea; supark@cnu.ac.kr (S.U.P.)

***** Correspondence: [swd2002@korea.kr](mailto:swd2002@korea.kr) (W.D.S.); Tel.: +82-63-238-5305; [kjkpj@inu.ac.kr](mailto:kjkpj@inu.ac.kr) (J.K.K.); Tel.: +82-32-835-8241

**^†^** These authors contributed equally to this work

Supplementary tables

**Table S1**. Relative retention times (RRT) and mass spectral data of hydrophilic compounds as trimethylsilyl derivatives.

**Table S2**. Relative retention times (RRT) and mass spectral data of lipophilic compounds as trimethylsilyl derivatives.

**Table S3**. Relative retention times (RRT) and concentration of fatty acid methyl esters (FAME) mixture and fatty acids.

**Table S4**. Composition and content (ratio/g) of hydrophilic compounds in perilla (Perilla frutescens) cultivars. **Table S5**. Composition and content (ratio/g) of hydrophilic compounds in sesame (Sesamum indicum) cultivars.

**Table S6**. Composition and content (µg/g) of policosanol compounds in perilla (Perilla frutescens) cultivars.

**Table S7**. Composition and content (µg/g) of policosanol compounds in sesame (Sesamum indicum) cultivars.

**Table S8**. Composition and content (µg/g) of sterol and terpenoid compounds in perilla (Perilla frutescens) cultivars.

**Table S9**. Composition and content (µg/g) of sterol and terpenoid compounds in sesame (Sesamum indicum) cultivars.

**Table S10**. Composition and content (mg/g) of fatty acids in perilla (Perilla frutescens) cultivars.

**Table S11**. Composition and content (mg/g) of fatty acids in sesame (Sesamum indicum) cultivars.

**Table S12**. OPLS-DA loading plots and VIP values of variables of perilla (Perilla frutescens) cultivars.

**Table S13**. OPLS-DA loading plots and VIP values of variables of sesame (Sesamum indicum) cultivars.

Table S1. Relative retention times (RRT) and mass spectral data of hydrophilic compounds as trimethylsilyl derivatives.

| Compound | RT ^a^ | RRT ^b^ | RI ^c^ | Quantification ion | Ref ion |
| --- | --- | --- | --- | --- | --- |
| Lactic acid | 7.998 | 0.444 | 1061 | 219 | 191 |
| Glycolic acid | 8.250 | 0.458 | 1074 | 205 | 177 |
| Alanine | 8.861 | 0.492 | 1106 | 190 | 147 |
| Valine | 10.888 | 0.604 | 1222 | 218 | 144 |
| Ethanolamine | 11.195 | 0.621 | 1241 | 174 | 100, 147 |
| Leucine | 11.792 | 0.654 | 1278 | 232 | 218 |
| Glycerol | 11.809 | 0.655 | 1279 | 218 | 205 |
| Phosphoric acid | 11.825 | 0.656 | 1280 | 314 | 299 |
| Isoleucine | 12.163 | 0.675 | 1301 | 232 | 218 |
| Nicotinic acid | 12.207 | 0.677 | 1304 | 180 | 106, 136 |
| Succinic acid | 12.340 | 0.684 | 1313 | 247 | 172 |
| Glycine | 12.384 | 0.687 | 1316 | 248.1 | 174 |
| Glyceric acid | 12.724 | 0.706 | 1339 | 292 | 189 |
| Fumaric acid | 12.812 | 0.711 | 1345 | 245 | 217 |
| Serine | 13.137 | 0.729 | 1367 | 306 | 278 |
| Threonine | 13.565 | 0.752 | 1396 | 291 | 218 |
| Beta-alanine | 14.117 | 0.783 | 1436 | 174 | 147, 248 |
| Malic acid | 14.937 | 0.829 | 1496 | 233 | 189 |
| Aspartic acid | 15.361 | 0.852 | 1529 | 334 | 306 |
| Methionine | 15.374 | 0.853 | 1530 | 293 | 250 |
| 4-Aminobutyric acid | 15.513 | 0.860 | 1541 | 304.1 | 174.1 |
| Threonic acid | 15.933 | 0.884 | 1574 | 292.2 |  |
| Glutamic acid | 16.600 | 0.921 | 1628 | 363 | 348 |
| Phenylalanine | 16.768 | 0.930 | 1642 | 218 | 192 |
| Ribitol (IS^d^) | 18.028 | 1.000 | 1750 | 319.1 | 307.1 |
| Glutamine | 18.423 | 1.022 | 1785 | 362 | 347 |
| Citric acid | 19.011 | 1.055 | 1839 | 363 | 347 |
| Isocitric acid | 19.011 | 1.055 | 1839 | 465 | 375 |
| Quinic acid | 19.494 | 1.081 | 1884 | 345 | 147, 255 |
| Fructose (1) | 19.707 | 1.093 | 1904 | 307 | 277.1 |
| Fructose (2) | 19.788 | 1.098 | 1912 | 307.1 | 277.1 |
| Glucose (1) | 19.972 | 1.108 | 1930 | 319.1 | 205.1 |
| Glucose (2) | 20.186 | 1.120 | 1951 | 319.1 | 205.1 |
| Inositol | 21.958 | 1.218 | 2132 | 318.1 | 305.1 |
| Tryptophan | 23.046 | 1.278 | 2251 | 405 | 377 |
| Sucrose | 26.802 | 1.487 | 2705 | 437.2 | 361.1 |
| Trehalose | 27.779 | 1.541 | 2812 | 361.1 | 271.1 |

^a^ Retention time (min)

^b^ Relative retention times (retention time of analyte/retention time of ribitol)

^c^ Retention index

^d^ IS, internal standard

| Compound | RT ^a^ | RRT ^b^ | [M]^+^ | Other characteristic ions ^c^, m/z (%) |
| --- | --- | --- | --- | --- |
| C20-ol | 10.28 | 0.782 | 370 (0) | **355 (100)** |
| C21-ol | 10.82 | 0.823 | 384 (0) | **369 (100)** |
| C22-ol | 11.33 | 0.862 | 398 (0) | **383 (100)** |
| C23-ol | 11.83 | 0.901 | 411 (0) | **397 (100)** |
| C24-ol | 12.33 | 0.936 | 426 (0) | **411 (100)** |
| C26-ol | 13.23 | 1.006 | 454 (0) | **439 (100)** |
| β-Tocopherol | 13.59 | 1.034 | 489(100) | 263 (13) **222 (87)** |
| γ-Tocopherol | 13.63 | 1.037 | 488 (60) | 263 (10) **223 (100)** |
| C27-ol | 13.68 | 1.041 | 468 (0) | **453 (100)** |
| C28-ol | 14.16 | 1.079 | 482 (0) | **467 (100)** |
| α-Tocopherol | 14.40 | 1.098 | 503 (90) | 277 (8) **237 (100)** |
| Campesterol | 15.25 | 1.166 | 473 (0) | 382 (38) 367 (23) **343 (50)** 255 (18) 129 (100) |
| C30-ol | 15.31 | 1.169 | 510 (0) | **496 (100),** 495 (60) |
| Stigmasterol | 15.44 | 1.181 | 484 (23) | **394 (38)** 355 (15) 255 (52) 129 (100) |
| β-Sitosterol | 15.86 | 1.214 | 487 (17) | 396 (42) **357 (44)** 255 (17) 129 (100) |
| β-Amyrin | 16.10 | 1.234 | 499 (0) | **218 (100)** 203 (42) 189 (26) |
| α-Amyrin | 16.39 | 1.259 | 499 (0) | 218 (100) 203 (18) **189 (32)** |

Table S2. Relative retention times (RRT) and mass spectral data of lipophilic compounds as trimethylsilyl derivatives.

^a^ Retention time (min)

^b^ Relative retention times (retention time of analyte/retention time of 5α-cholestane)

^c^ Ions in boldface indicate the specific mass ion used for quantification

Table S3. Relative retention times (RRT) and concentration of fatty acid methyl esters (FAME) mixture and fatty acids

| No. | Methylated  standard mixture | Compound name | C | RT^a^ | RRT^b^ | Area | Weight% | ppm | µg | Ratio^c^ |
| --- | --- | --- | --- | --- | --- | --- | --- | --- | --- | --- |
| 1 | Methyl caprylate | Caprylic acid | C8:0 | 12.83 | 0.47 | 120581 | 8 | 8 | 0.8 | 2.38 |
| 2 | Methyl caprate | Capric acid | C10:0 | 17.59 | 0.64 | 72893 | 8 | 8 | 0.8 | 1.44 |
| 3 | Methyl laurate | Lauric acid | C12:0 | 21.85 | 0.80 | 31987 | 8 | 8 | 0.8 | 0.63 |
| 4 | Methyl myristate | Myristic acid | C14:0 | 25.67 | 0.94 | 47284 | 8 | 8 | 0.8 | 0.93 |
| 5 | IS | Pentadecanoic acid | C15:0 | 27.44 | 1.00 | 50674 |  | 10 | 1 | 1.00 |
| 6 | Methyl palmitoleate | Palmitoleic acid | C16:1n7 | 28.79 | 1.05 | 4795 | 5 | 5 | 0.5 | 0.09 |
| 7 | Methyl palmitate | Palmitic acid | C16:0 | 29.13 | 1.06 | 104565 | 11 | 11 | 1.1 | 2.06 |
| 8 | Methyl linoleate | Linoleic acid | C18:2n6 | 31.82 | 1.16 | 6577 | 5 | 5 | 0.5 | 0.13 |
| 9 | Methyl linolenate | α-Linolenic acid | C18:3n3 | 31.93 | 1.16 | 3244 | 5 | 5 | 0.5 | 0.06 |
| 10 | Methyl oleate | Oleic acid | C18:1n9 | 31.90 | 1.16 | 4515 | 5 | 5 | 0.5 | 0.09 |
| 11 | Methyl stearate | Stearic acid | C18:0 | 32.29 | 1.18 | 87478 | 8 | 8 | 0.8 | 1.73 |
| 12 | Methyl arachisate | Arachidic acid | C20:0 | 35.18 | 1.28 | 85959 | 8 | 8 | 0.8 | 1.70 |
| 13 | Methyl erucate | Erucic acid | C22:1n9 | 37.55 | 1.37 | 18996 | 5 | 5 | 0.5 | 0.37 |
| 14 | Methyl behenate | Behenic acid | C22:0 | 37.86 | 1.38 | 90082 | 8 | 8 | 0.8 | 1.78 |
| 15 | Methyl lignocerate | Lignoceric acid | C24:0 | 40.34 | 1.47 | 70579 | 8 | 8 | 0.8 | 1.39 |

^a^ Retention time (min)

^b^ Relative retention times (retention time of analyte/retention time of pentadecanoic acid)

^b^ Ratio (area of analyte/area of pentadecanoic acid)

Table S4. Composition and content (ratio/g) of hydrophilic compounds in perilla (*Perilla frutescens*) cultivars.

| Compound^a^ | Lactic acid | Glycolic acid | Alanine | Valine | Ethanolamine | Leucine | Glycerol | Phosphoric acid | Isoleucine | Proline |
| --- | --- | --- | --- | --- | --- | --- | --- | --- | --- | --- |
| KP1 | 0.42±0.03 | 0.14±0.01 | 0.84±0.11 | 2.20±0.03 | 1.16±0.09 | 0.30±0.01 | 8.30±7.91 | 11.76±0.85 | 0.29±0.01 | ND |
| KP2 | 0.54±0.09 | ND^b^ | 0.86±0.29 | 2.70±0.41 | 1.31±0.18 | 0.45±0.07 | 7.68±5.09 | 17.36±5.97 | 0.24±0.05 | ND |
| KP3 | 0.56±0.02 | ND | 0.99±0.05 | 2.50±0.44 | 1.37±0.07 | 0.46±0.10 | 20.86±9.66 | 14.35±1.27 | 0.26±0.07 | ND |
| KP4 | 0.60±0.14 | 0.23±0.03 | 1.36±0.08 | 2.04±0.05 | 1.40±0.05 | 0.29±0.02 | 9.09±1.44 | 16.00±0.28 | 0.21±0.01 | ND |
| CP1 | 0.52±0.11 | 0.31±0.01 | 0.52±0.12 | 0.66±0.11 | 1.34±0.12 | ND | 60.32±17.05 | 16.01±2.54 | ND | ND |
| CP2 | 0.60±0.06 | 0.32±0.05 | 0.67±0.15 | 2.61±0.26 | 1.28±0.10 | 0.57±0.04 | 14.49±0.23 | 16.41±1.53 | 0.35±0.05 | ND |
| CP3 | 0.37±0.03 | 0.43±0.06 | 1.38±0.04 | 4.38±0.45 | 1.36±0.08 | 0.83±0.10 | 2.95±0.18 | 22.96±3.76 | 0.32±0.03 | ND |
| CP4 | 0.77±0.05 | 0.53±0.03 | 2.65±0.25 | 2.08±0.08 | 1.32±0.08 | 0.39±0.01 | 53.47±4.66 | 21.09±1.57 | 0.30±0.01 | ND |
| CP5 | 0.64±0.17 | 0.43±0.04 | 0.91±0.15 | 1.80±0.17 | 1.40±0.15 | 0.56±0.08 | 3.97±1.51 | 14.16±0.72 | 0.25±0.02 | ND |
| CP6 | 0.78±0.13 | 1.01±0.06 | 1.30±0.24 | 4.40±0.84 | 1.44±0.16 | 0.64±0.10 | 9.10±1.58 | 40.44±5.9 | 0.40±0.11 | ND |
| CP7 | 0.88±0.04 | 0.50±0.07 | 3.34±1.66 | 5.73±2.03 | 1.47±0.10 | 0.87±0.19 | 9.87±1.39 | 31.55±1.06 | 0.55±0.14 | ND |
| CP8 | 0.75±0.18 | 0.39±0.04 | 0.82±0.15 | 2.91±0.59 | 1.38±0.12 | 0.41±0.03 | 61.25±21.85 | 22.9±2.04 | 0.32±0.08 | ND |
| CP9 | 0.82±0.10 | 0.40±0.03 | 1.18±0.08 | 2.80±0.20 | 1.42±0.10 | 0.43±0.03 | 11.25±1.56 | 24.97±1.21 | 0.36±0.06 | ND |
| CP10 | 1.06±0.12 | 0.57±0.08 | 0.98±0.12 | 2.77±0.43 | 1.82±0.13 | 0.39±0.02 | 9.27±1.18 | 23.25±0.99 | 0.32±0.06 | ND |
| CP11 | 0.68±0.03 | 0.48±0.04 | 1.47±0.16 | 2.49±0.10 | 1.24±0.09 | 0.48±0.02 | 12.68±2.01 | 31.19±1.29 | 0.32±0.01 | ND |
| CP12 | 0.61±0.06 | 0.29±0.03 | 1.06±0.20 | 3.02±0.22 | 1.12±0.09 | 0.62±0.03 | 62.30±37.14 | 17.64±2.17 | 0.34±0.06 | ND |
| CP13 | 0.92±0.11 | 0.35±0.03 | 0.49±0.14 | 2.36±0.41 | 1.36±0.14 | 0.41±0.08 | 6.79±4.25 | 10.93±4.43 | 0.28±0.05 | ND |
| CP14 | 0.94±0.18 | 0.44±0.04 | 1.22±0.27 | 1.74±0.10 | 1.30±0.14 | 0.34±0.04 | 120.34±23.4 | 33.66±0.98 | 0.26±0.04 | ND |
| CP15 | 0.99±0.19 | 0.42±0.10 | 1.62±0.35 | 3.21±0.39 | 1.65±0.18 | 0.43±0.06 | 13.91±4.60 | 29.64±3.35 | 0.30±0.03 | ND |

(*Continued next page*)

(*Table S4., continued*)

| Compound^a^ | Nicotinic acid | Succinic acid | Glycine | Glyceric acid | Fumaric acid | Serine | Threonine | β-Alanine | Malic acid | Aspartic acid |
| --- | --- | --- | --- | --- | --- | --- | --- | --- | --- | --- |
| KP1 | 1.42±0.05 | 0.57±0.11 | 0.97±0.08 | 0.11±0.08 | 0.30±0.02 | 0.22±0.01 | 0.47±0.06 | 1.16±0.01 | 14.41±0.42 | 0.45±0.02 |
| KP2 | 1.72±0.11 | 0.45±0.25 | 1.66±0.14 | 0.37±0.21 | 0.60±0.41 | 0.24±0.06 | 0.62±0.09 | 1.33±0.19 | 18.83±2.89 | 0.35±0.05 |
| KP3 | 1.32±0.17 | 0.30±0.02 | 1.68±0.36 | 0.48±0.17 | 0.85±0.24 | 0.26±0.03 | 0.64±0.16 | 1.04±0.05 | 8.65±1.15 | 0.18±0.02 |
| KP4 | 2.18±0.15 | 1.03±0.02 | 1.38±0.05 | 0.61±0.05 | 0.92±0.12 | 0.23±0.02 | 0.60±0.02 | 1.17±0.01 | 34.2±0.44 | 0.49±0.03 |
| CP1 | 1.41±0.19 | 0.51±0.08 | 1.21±0.20 | 0.84±0.20 | 2.74±1.49 | 0.07±0.01 | 0.17±0.03 | 0.92±0.07 | 4.31±0.65 | 0.11±0.01 |
| CP2 | 2.10±0.22 | 0.45±0.03 | 1.23±0.15 | 0.52±0.11 | 0.85±0.08 | 0.31±0.02 | 0.63±0.11 | 0.99±0.13 | 6.22±0.19 | 0.63±0.07 |
| CP3 | 1.86±0.13 | 0.98±0.21 | 1.39±0.99 | 1.08±0.26 | 1.51±0.33 | 1.36±0.20 | 0.53±0.07 | 2.27±0.14 | 18.94±2.40 | 1.29±0.20 |
| CP4 | 2.11±0.01 | 1.13±0.07 | 1.73±0.13 | 1.57±0.26 | 5.64±0.80 | 0.24±0.03 | 0.44±0.05 | 1.08±0.03 | 10.88±4.03 | 0.27±0.03 |
| CP5 | 2.08±0.07 | 0.45±0.05 | 1.60±0.14 | 0.69±0.04 | 0.35±0.01 | 0.29±0.04 | 0.28±0.02 | 1.24±0.14 | 7.77±0.56 | 0.49±0.01 |
| CP6 | 1.59±0.16 | 2.06±0.12 | 3.14±0.54 | 3.09±0.34 | 1.51±0.21 | 1.01±0.14 | 0.66±0.16 | 2.88±0.12 | 11.87±2.10 | 0.66±0.16 |
| CP7 | 2.10±0.20 | 1.35±0.16 | 4.53±1.12 | 3.02±1.24 | 1.83±0.27 | 1.37±0.42 | 0.89±0.27 | 3.93±0.99 | 20.35±2.11 | 0.65±0.05 |
| CP8 | 2.37±0.12 | 0.62±0.02 | 2.71±0.28 | 0.49±0.12 | 5.01±1.47 | 0.13±0.01 | 0.22±0.02 | 1.16±0.13 | 9.13±0.83 | 0.48±0.08 |
| CP9 | 2.34±0.18 | 0.88±0.04 | 1.95±0.04 | 0.70±0.04 | 1.48±0.16 | 0.34±0.07 | 0.39±0.11 | 1.01±0.09 | 6.33±0.75 | 0.41±0.06 |
| CP10 | 3.26±0.18 | 0.94±0.18 | 1.69±0.26 | 0.75±0.25 | 2.78±0.46 | 0.42±0.01 | 0.44±0.12 | 1.26±0.11 | 17.29±0.77 | 0.85±0.05 |
| CP11 | 2.99±0.12 | 0.80±0.02 | 2.05±0.06 | 0.64±0.08 | 2.77±0.05 | 0.40±0.02 | 0.29±0.03 | 1.11±0.08 | 8.19±0.32 | 0.37±0.06 |
| CP12 | 1.71±0.05 | 0.92±0.47 | 1.63±0.22 | 0.50±0.03 | 18.82±21.66 | 0.50±0.10 | 0.52±0.07 | 1.20±0.07 | 13.47±2.25 | 0.53±0.06 |
| CP13 | 2.11±0.16 | 0.25±0.03 | 1.09±0.25 | 0.22±0.04 | 0.64±0.05 | 0.27±0.06 | 0.45±0.15 | 1.17±0.12 | 9.85±3.18 | 0.88±0.16 |
| CP14 | 2.51±0.09 | 2.70±0.16 | 2.66±0.27 | 1.25±0.12 | 54.28±13.87 | 0.25±0.01 | 0.25±0.03 | 1.24±0.16 | 30.37±4.90 | 0.36±0.02 |
| CP15 | 2.49±0.22 | 1.67±0.43 | 2.00±0.35 | 1.33±0.35 | 4.88±1.38 | 0.42±0.06 | 0.49±0.10 | 1.40±0.18 | 21.80±3.64 | 0.66±0.10 |

(*Continued next page*)

(*Table S4., continued*)

| Compound^a^ | Methionine | 4-Aminobutyric acid | Threonic acid | Glutamic acid | Phenylalanine | Glutamine | Xylose | Galactose | Citric acid | Isocitric acid |
| --- | --- | --- | --- | --- | --- | --- | --- | --- | --- | --- |
| KP1 | 0.09±0.01 | 0.87±0.18 | 1.33±0.13 | 0.57±0.01 | 12.55±0.60 | ND | ND | ND | 19.57±0.84 | 10.27±0.56 |
| KP2 | 0.13±0.04 | 1.58±1.01 | 2.05±1.13 | 0.33±0.08 | 14.37±1.66 | 0.46±0.2 | ND | ND | 27.92±6.75 | 14.36±3.73 |
| KP3 | 0.14±0.04 | 0.85±0.28 | 1.11±0.11 | 0.25±0.01 | 12.15±0.70 | 0.30±0.01 | ND | ND | 12.96±2.74 | 6.51±1.34 |
| KP4 | 0.14±0.01 | 1.44±0.25 | 3.99±0.22 | 0.45±0.02 | 16.23±0.06 | 0.32±0.05 | ND | ND | 49.29±0.20 | 25.47±0.34 |
| CP1 | ND | 1.83±0.67 | 0.80±0.35 | 0.11±0.01 | 6.29±0.80 | ND | ND | ND | 2.61±0.57 | 1.37±0.28 |
| CP2 | ND | 1.87±0.29 | 2.20±0.40 | 0.77±0.08 | 17.64±1.84 | ND | ND | ND | 22.33±1.67 | 11.77±0.82 |
| CP3 | 0.21±0.05 | 1.49±0.20 | 6.51±3.00 | 0.76±0.06 | 16.15±0.96 | 0.69±0.03 | ND | ND | 30.69±2.72 | 15.7±1.19 |
| CP4 | 0.05±0.02 | 5.13±0.50 | 1.97±0.19 | 0.36±0.03 | 14.55±0.36 | ND | ND | ND | 19.4±0.88 | 9.71±0.50 |
| CP5 | 0.04±0.01 | 0.42±0.07 | 2.10±0.21 | 0.34±0.02 | 15.93±1.32 | ND | ND | ND | 12.48±0.48 | 6.43±0.23 |
| CP6 | 0.03±0.04 | 3.95±0.80 | 11.28±2.07 | 0.62±0.12 | 16.61±2.27 | 0.33±0.04 | ND | ND | 18.56±3.48 | 9.7±1.71 |
| CP7 | 0.11±0.02 | 4.92±1.25 | 5.50±1.16 | 0.61±0.06 | 18.18±2.93 | 0.33±0.06 | ND | ND | 26.68±3.07 | 14.18±1.54 |
| CP8 | 0.05±0.00 | 2.30±0.43 | 2.04±0.10 | 0.31±0.03 | 12.95±1.37 | 0.08±0.12 | ND | ND | 14.99±1.72 | 7.85±0.75 |
| CP9 | 0.05±0.01 | 5.85±0.56 | 2.29±0.23 | 0.51±0.06 | 14.17±0.21 | ND | ND | ND | 19.83±0.36 | 10.15±0.14 |
| CP10 | 0.07±0.01 | 2.09±0.54 | 6.52±2.84 | 0.48±0.05 | 16.64±1.40 | ND | ND | ND | 39.13±2.44 | 19.86±1.30 |
| CP11 | 0.06±0.02 | 2.04±0.25 | 2.96±0.10 | 0.69±0.02 | 14.84±0.46 | ND | ND | ND | 26.94±1.38 | 13.62±1.10 |
| CP12 | 0.15±0.01 | 2.87±1.01 | 2.52±0.22 | 0.57±0.11 | 15.21±1.52 | 0.29±0.10 | ND | ND | 20.74±1.81 | 10.46±1.08 |
| CP13 | 0.04±0.01 | 0.28±0.06 | 2.13±0.32 | 0.42±0.04 | 12.91±1.20 | 0.27±0.04 | ND | ND | 16.71±2.09 | 8.52±1.28 |
| CP14 | 0.10±0.02 | 5.66±0.27 | 3.93±0.26 | 0.29±0.01 | 13.64±0.23 | ND | ND | ND | 29.06±1.10 | 14.62±0.59 |
| CP15 | 0.10±0.02 | 3.76±0.73 | 12.47±5.72 | 0.54±0.08 | 16.18±2.05 | ND | ND | ND | 26.36±4.58 | 13.54±2.29 |

(*Continued next page*)

(*Table S4., continued*)

| Compound^a^ | Quinic acid | Fructose | Glucose | Mannitol | Inositol | Tryptophan | Sucrose | Trehalose |
| --- | --- | --- | --- | --- | --- | --- | --- | --- |
| KP1 | ND | 3.61±2.18 | 16.31±1.52 | 3.58±0.89 | 4.76±0.30 | 0.40±0.08 | 328.19±11.75 | 8.17±0.28 |
| KP2 | 1.29±1.32 | 8.22±6.66 | 38.64±26.29 | 1.77±0.33 | 7.03±1.48 | 0.24±0.05 | 333.05±56.81 | 7.13±2.17 |
| KP3 | ND | 21.52±16.34 | 26.10±13.77 | 6.04±1.14 | 5.57±2.12 | 0.16±0.03 | 281.84±20.14 | 3.64±0.28 |
| KP4 | 7.59±0.66 | 8.99±2.34 | 52.69±1.79 | 9.24±1.26 | 20.39±1.09 | 0.18±0.05 | 341.18±14.48 | 6.40±0.16 |
| CP1 | ND | 9.90±5.79 | 14.15±4.11 | 19.43±4.64 | 2.59±0.69 | ND | 244.82±37.63 | 2.69±0.98 |
| CP2 | 1.90±0.31 | 9.71±1.19 | 37.48±5.81 | 6.11±1.57 | 4.05±0.45 | 0.23±0.00 | 346.64±26.65 | 4.07±0.28 |
| CP3 | 4.27±3.29 | 101.22±68.86 | 187.48±80.79 | 2.01±1.03 | 16.44±4.76 | 0.18±0.02 | 362.11±10.01 | 7.90±0.54 |
| CP4 | 2.20±0.42 | 28.78±11.87 | 68.49±14.00 | 10.01±1.93 | 5.67±0.56 | 0.27±0.06 | 316.23±9.60 | 3.42±0.14 |
| CP5 | 0.55±0.06 | 6.58±1.29 | 39.22±3.43 | 1.97±0.17 | 4.00±0.15 | 0.25±0.02 | 331.02±19.94 | 3.85±0.32 |
| CP6 | 2.24±0.46 | 55.22±7.21 | 229.98±17.62 | 1.18±0.36 | 27.71±5.68 | 0.03±0.04 | 370.5±36.86 | 4.17±0.65 |
| CP7 | 3.35±0.94 | 68.23±10.04 | 163.62±26.40 | 2.89±0.33 | 14.42±0.80 | 0.17±0.01 | 388.28±21.20 | 7.84±1.49 |
| CP8 | 0.36±0.02 | 35.34±15.33 | 52.77±15.73 | 10.63±2.84 | 8.20±1.56 | 0.24±0.04 | 386.04±28.42 | 5.73±0.57 |
| CP9 | 4.78±0.66 | 30.79±2.53 | 78.65±5.49 | 1.49±0.09 | 8.01±0.07 | 0.24±0.01 | 366.36±8.44 | 3.32±0.16 |
| CP10 | ND | 28.91±14.13 | 38.30±9.42 | 4.62±0.47 | 11.00±2.57 | 0.37±0.05 | 418.86±28.21 | 6.15±0.27 |
| CP11 | 0.41±0.02 | 26.07±7.35 | 46.25±8.64 | 6.36±1.83 | 7.75±0.97 | 0.53±0.08 | 430.83±6.54 | 5.22±0.28 |
| CP12 | 0.45±0.12 | 17.5±0.98 | 39.76±2.73 | 10.56±4.45 | 6.64±1.15 | 0.31±0.04 | 369.19±9.43 | 4.35±0.54 |
| CP13 | ND | 2.44±0.38 | 11.97±1.22 | 3.92±2.79 | 5.07±1.83 | 0.46±0.02 | 396.77±29.54 | 4.98±0.96 |
| CP14 | 0.30±0.19 | 48.27±4.98 | 44.94±2.38 | 22.87±2.45 | 15.63±2.46 | 0.25±0.01 | 396.93±26.12 | 5.35±0.11 |
| CP15 | 1.05±0.48 | 35.60±3.81 | 44.38±2.99 | 3.05±1.25 | 18.93±6.33 | 0.31±0.05 | 530.02±52.64 | 6.50±0.05 |

^a^ Each value is the mean of three replications ± standard deviation. ^b^ ND, Not detected.Table S5-Composition and content (ratio/g) of hydrophilic compounds in sesame (*Sesamum indicum*) cultivars

| Compound^a^ | Lactic acid | Glycolic acid | Alanine | Valine | Ethanolamine | Leucine | Glycerol | Phosphoric acid | Isoleucine | Proline |
| --- | --- | --- | --- | --- | --- | --- | --- | --- | --- | --- |
| KS1 | 1.86±0.16 | 6.61±0.40 | 1.42±0.19 | 3.35±0.21 | ND^b^ | 1.31±0.03 | 6.82±0.79 | 36.28±2.50 | 1.15±0.06 | 1.09±0.08 |
| KS2 | 2.30±1.30 | 5.63±0.52 | 2.47±0.48 | 4.84±0.86 | ND | 1.53±0.25 | 5.61±0.34 | 40.06±5.78 | 1.37±0.23 | 2.13±0.13 |
| KS3 | 1.53±0.25 | 6.03±0.15 | 1.83±0.11 | 3.71±0.20 | ND | 1.38±0.07 | 11.76±2.4 | 37.44±1.74 | 1.10±0.12 | 1.29±0.10 |
| KS4 | 3.59±2.84 | 5.43±0.19 | 1.57±0.20 | 4.51±0.21 | ND | 1.48±0.04 | 6.15±1.95 | 32.43±2.24 | 1.46±0.05 | 1.20±0.07 |
| KS5 | 1.58±0.70 | 5.89±0.27 | 1.95±0.25 | 5.76±0.2 | ND | 1.79±0.12 | 6.10±1.11 | 40.39±0.42 | 1.68±0.02 | 1.88±0.08 |
| CS1 | 2.29±1.27 | 7.19±0.48 | 2.47±0.24 | 6.20±0.38 | ND | 1.76±0.04 | 25.78±3.47 | 42.81±3.29 | 1.46±0.04 | 0.57±0.05 |
| CS2 | 2.45±1.85 | 6.70±0.69 | 2.81±0.71 | 6.70±0.30 | ND | 1.76±0.12 | 23.21±4.37 | 45.07±1.44 | 1.60±0.07 | 0.73±0.05 |
| CS3 | 6.76±5.08 | 7.01±0.22 | 5.08±0.36 | 9.06±1.80 | ND | 2.15±0.21 | 25.42±5.33 | 46.56±4.39 | 1.97±0.24 | 1.01±0.12 |
| CS4 | 6.53±2.98 | 6.52±0.26 | 6.24±0.41 | 5.08±0.14 | ND | 1.23±0.02 | 34.13±4.46 | 59.27±6.78 | 1.33±0.05 | 0.74±0.08 |
| CS5 | 0.92±0.19 | 6.26±0.84 | 5.34±0.84 | 3.55±0.40 | ND | 1.14±0.04 | 20.23±1.32 | 45.72±0.64 | 0.90±0.15 | 0.60±0.07 |
| CS6 | 0.76±0.17 | 5.46±0.29 | 4.99±0.09 | 4.14±0.18 | ND | 1.30±0.06 | 20.77±0.71 | 49.95±2.17 | 1.06±0.04 | 0.50±0.02 |
| CS7 | 1.00±0.06 | 5.21±0.06 | 4.76±1.17 | 3.43±0.40 | ND | 1.21±0.08 | 25.32±2.90 | 44.24±2.91 | 0.88±0.12 | 0.38±0.02 |
| CS8 | 0.88±0.19 | 4.66±0.24 | 4.98±0.64 | 3.44±0.21 | ND | 1.16±0.02 | 22.23±3.60 | 47.65±2.86 | 0.93±0.07 | 0.37±0.05 |
| CS9 | 0.89±0.21 | 4.73±0.57 | 2.68±0.34 | 4.51±0.21 | ND | 1.57±0.06 | 12.97±1.20 | 39.7±5.57 | 1.27±0.10 | 0.52±0.08 |
| CS10 | 0.53±0.09 | 5.81±0.29 | 2.21±0.41 | 4.69±0.28 | ND | 1.76±0.02 | 10.55±0.83 | 34.65±3.07 | 1.29±0.11 | 0.73±0.20 |
| CS11 | 0.92±0.41 | 5.06±0.47 | 5.67±0.61 | 4.31±0.18 | ND | 1.76±0.08 | 13.69±0.59 | 39.51±3.93 | 1.41±0.08 | 0.57±0.05 |
| CS12 | 1.10±0.25 | 4.96±0.40 | 6.92±1.36 | 3.40±0.39 | ND | 1.07±0.21 | 41.55±13.05 | 53.44±2.88 | 0.80±0.09 | 0.94±0.38 |
| CS13 | 0.94±0.13 | 5.16±0.16 | 5.68±2.40 | 3.16±0.22 | ND | 1.33±0.02 | 13.86±1.12 | 35.94±2.10 | 0.90±0.12 | 0.43±0.03 |
| CS14 | 1.27±0.10 | 5.44±0.35 | 4.62±0.80 | 3.19±0.45 | ND | 1.27±0.07 | 29.02±5.86 | 50.28±1.90 | 0.80±0.06 | 1.16±0.47 |
| CS15 | 1.19±0.51 | 4.87±0.15 | 2.82±0.25 | 4.32±0.58 | ND | 1.39±0.26 | 81.39±6.78 | 47.9±3.10 | 1.16±0.21 | 0.18±0.01 |
| CS16 | 0.77±0.27 | 5.18±0.09 | 3.96±1.09 | 5.33±0.43 | ND | 1.55±0.08 | 91.72±29.37 | 49.95±1.68 | 1.42±0.03 | 0.28±0.04 |
| CS17 | 0.86±0.14 | 4.33±0.32 | 3.25±0.74 | 3.54±0.23 | ND | 1.37±0.18 | 51.83±24.13 | 45.19±4.00 | 0.97±0.13 | 0.29±0.12 |
| CS18 | 1.17±0.19 | 5.00±0.42 | 3.74±0.27 | 3.61±0.13 | ND | 1.29±0.10 | 18.09±1.36 | 49.47±2.72 | 0.99±0.09 | 0.67±0.07 |
| CS19 | 1.06±0.16 | 4.60±0.27 | 3.03±0.95 | 4.47±0.46 | ND | 1.61±0.16 | 34.72±30.34 | 39.75±1.56 | 1.23±0.16 | 0.56±0.02 |
| CS20 | 0.70±0.05 | 4.59±0.46 | 2.47±0.51 | 4.45±0.56 | ND | 1.60±0.19 | 12.14±0.69 | 36.10±3.01 | 1.22±0.16 | 0.60±0.11 |
| CS21 | 0.93±0.19 | 4.09±0.64 | 2.26±0.61 | 3.72±0.44 | ND | 1.43±0.11 | 17.66±1.37 | 29.44±1.68 | 1.01±0.15 | 0.47±0.11 |

(*Continued next page*)

(*Table S5., continued*)

| Compound^a^ | Nicotinic acid | Succinic acid | Glycine | Glyceric acid | Fumaric acid | Serine | Threonine | β-Alanine | Malic acid | Aspartic acid |
| --- | --- | --- | --- | --- | --- | --- | --- | --- | --- | --- |
| KS1 | ND | 1.53±0.18 | 2.29±0.12 | 0.51±0.05 | 4.02±0.37 | 0.66±0.04 | 1.45±0.1 | ND | 66.17±3.99 | 1.12±0.08 |
| KS2 | ND | 1.84±0.29 | 3.30±0.60 | 0.68±0.16 | 5.61±1.10 | 0.82±0.19 | 2.70±0.26 | ND | 74.39±11.53 | 1.40±0.19 |
| KS3 | ND | 1.55±0.06 | 4.09±0.16 | 0.94±0.11 | 4.29±0.13 | 0.68±0.06 | 2.72±0.18 | ND | 73.35±5.04 | 2.28±0.08 |
| KS4 | ND | 1.23±0.02 | 2.86±1.03 | 0.66±0.11 | 3.58±0.35 | 0.70±0.21 | 2.38±0.26 | ND | 65.18±4.01 | 1.67±0.11 |
| KS5 | ND | 1.51±0.17 | 3.09±0.38 | 0.81±0.06 | 5.74±0.44 | 0.64±0.06 | 2.67±0.05 | ND | 89.27±3.93 | 1.51±0.07 |
| CS1 | ND | 2.13±0.33 | 5.43±0.35 | 0.95±0.15 | 5.19±0.09 | 1.09±0.12 | 2.60±0.05 | ND | 70.93±2.76 | 2.07±0.21 |
| CS2 | ND | 2.74±0.2 | 6.84±0.36 | 1.43±0.34 | 5.89±0.51 | 1.21±0.14 | 2.98±0.20 | ND | 73.46±6.84 | 2.26±0.12 |
| CS3 | ND | 4.62±0.57 | 8.63±0.60 | 1.47±0.25 | 9.24±1.57 | 1.82±0.32 | 2.94±0.19 | ND | 95.92±1.08 | 1.76±0.35 |
| CS4 | ND | 4.64±0.51 | 10.92±0.60 | 2.34±0.99 | 11.88±0.72 | 1.35±0.23 | 2.84±0.32 | ND | 61.42±3.05 | 1.85±0.12 |
| CS5 | ND | 3.23±0.57 | 6.48±0.26 | 1.12±0.07 | 7.37±0.38 | 0.72±0.09 | 1.47±0.21 | ND | 70.30±1.88 | 1.47±0.27 |
| CS6 | ND | 2.13±0.21 | 7.06±0.13 | 1.22±0.23 | 5.35±0.26 | 0.92±0.03 | 1.89±0.07 | ND | 60.22±4.02 | 1.42±0.13 |
| CS7 | ND | 2.15±0.36 | 6.72±1.37 | 1.46±0.72 | 5.31±1.12 | 0.69±0.05 | 1.63±0.33 | ND | 53.09±2.23 | 1.25±0.13 |
| CS8 | ND | 2.32±0.65 | 6.63±0.38 | 1.55±0.77 | 4.25±0.15 | 0.62±0.06 | 1.69±0.24 | ND | 50.59±5.35 | 1.33±0.19 |
| CS9 | ND | 1.81±0.19 | 3.84±0.28 | 0.79±0.10 | 3.04±0.51 | 0.76±0.02 | 1.93±0.23 | ND | 67.28±9.76 | 1.40±0.08 |
| CS10 | ND | 1.44±0.06 | 3.50±0.37 | 0.59±0.06 | 2.29±0.24 | 0.74±0.09 | 1.78±0.15 | ND | 55.80±3.80 | 1.33±0.22 |
| CS11 | ND | 3.04±0.49 | 5.83±0.18 | 0.96±0.13 | 3.17±1.24 | 0.63±0.09 | 1.93±0.10 | ND | 72.70±10.87 | 1.36±0.15 |
| CS12 | ND | 3.93±0.71 | 7.80±0.83 | 2.80±0.22 | 8.04±4.34 | 0.74±0.08 | 2.01±0.27 | ND | 65.70±3.70 | 1.00±0.04 |
| CS13 | ND | 4.33±1.93 | 5.59±1.12 | 1.25±0.41 | 3.36±0.45 | 0.62±0.10 | 1.55±0.15 | ND | 63.28±8.01 | 1.05±0.31 |
| CS14 | ND | 2.86±0.21 | 6.31±1.41 | 1.49±0.24 | 3.77±0.55 | 0.60±0.10 | 1.82±0.33 | ND | 52.27±5.09 | 0.95±0.14 |
| CS15 | ND | 1.68±0.31 | 6.13±0.62 | 0.98±0.27 | 4.97±0.51 | 0.48±0.07 | 1.75±0.19 | ND | 17.98±4.89 | 1.20±0.33 |
| CS16 | ND | 1.92±0.38 | 6.93±1.01 | 1.06±0.08 | 4.79±1.87 | 0.62±0.08 | 2.04±0.36 | ND | 31.98±7.88 | 1.37±0.13 |
| CS17 | ND | 2.61±1.33 | 4.95±0.76 | 1.04±0.11 | 19.63±11.43 | 0.49±0.16 | 1.25±0.06 | ND | 64.41±41.93 | 1.14±0.42 |
| CS18 | ND | 1.50±0.04 | 5.55±0.16 | 0.97±0.17 | 5.39±1.16 | 0.51±0.03 | 1.22±0.03 | ND | 61.90±5.37 | 1.56±0.02 |
| CS19 | ND | 1.81±0.38 | 4.31±0.37 | 0.96±0.15 | 3.21±0.48 | 0.65±0.06 | 1.61±0.14 | ND | 70.72±4.00 | 1.26±0.14 |
| CS20 | ND | 1.63±0.2 | 3.31±0.41 | 0.76±0.20 | 2.64±0.52 | 0.63±0.12 | 1.73±0.37 | ND | 64.04±11.51 | 1.25±0.23 |
| CS21 | ND | 1.48±0.39 | 3.20±0.23 | 0.67±0.07 | 2.27±0.49 | 0.46±0.08 | 1.38±0.24 | ND | 53.51±3.81 | 1.10±0.28 |

(*Continued next page*)

(*Table S5., continued*)

| Compound^a^ | Methionine | 4-Aminobutyric acid | Threonic acid | Glutamic acid | Phenylalanine | Glutamine | Xylose | Galactose | Citric acid | Isocitric acid |
| --- | --- | --- | --- | --- | --- | --- | --- | --- | --- | --- |
| KS1 | ND | 0.59±0.06 | 3.64±0.47 | 0.39±0.03 | 36.65±4.20 | ND | 4.79±0.72 | 107.68±7.90 | 15.3±0.90 | 7.35±0.61 |
| KS2 | ND | 1.36±0.59 | 3.78±1.05 | 0.36±0.04 | 35.80±4.47 | ND | 5.30±0.95 | 80.19±9.94 | 15.53±2.17 | 7.81±1.01 |
| KS3 | ND | 1.28±0.09 | 1.62±0.11 | 0.32±0.03 | 43.37±2.77 | ND | 2.57±0.60 | 102.96±1.00 | 13.38±1.01 | 6.47±0.67 |
| KS4 | ND | 0.61±0.06 | 2.04±0.33 | 0.39±0.04 | 47.62±4.45 | ND | 6.41±1.50 | 93.94±8.84 | 9.49±1.36 | 4.57±0.61 |
| KS5 | ND | 1.08±0.08 | 3.12±0.38 | 0.35±0.02 | 46.56±1.73 | ND | 8.16±1.28 | 110.87±3.05 | 14.86±0.68 | 7.25±0.47 |
| CS1 | ND | 1.69±0.27 | 3.73±0.26 | 0.55±0.04 | 82.72±2.79 | ND | 9.42±0.94 | 107.2±4.64 | 14.12±0.85 | 6.70±0.34 |
| CS2 | ND | 4.69±2.88 | 3.51±0.29 | 0.57±0.04 | 84.68±3.75 | ND | 9.13±0.89 | 98.36±5.68 | 18.18±1.44 | 8.68±0.66 |
| CS3 | ND | 3.87±2.42 | 2.37±0.25 | 0.48±0.05 | 76.40±10.66 | ND | 9.65±2.16 | 114.84±2.53 | 13.38±1.39 | 6.39±0.60 |
| CS4 | ND | 27.29±3.46 | 2.33±0.42 | 0.29±0.03 | 66.97±3.96 | ND | 3.11±0.84 | 100.03±4.86 | 12.18±0.41 | 5.90±0.19 |
| CS5 | ND | 7.02±1.32 | 1.65±0.17 | 0.27±0.05 | 58.09±5.19 | ND | 3.78±0.56 | 94.74±8.02 | 10.91±1.39 | 5.29±0.72 |
| CS6 | ND | 19.28±1.56 | 2.04±0.09 | 0.25±0.02 | 63.69±4.92 | ND | 2.89±0.72 | 100.82±3.32 | 9.06±0.82 | 4.27±0.32 |
| CS7 | ND | 19.2±5.88 | 1.51±0.29 | 0.20±0.01 | 55.69±7.67 | ND | 2.91±0.18 | 92.49±5.91 | 8.16±0.74 | 3.96±0.35 |
| CS8 | ND | 18.4±6.24 | 1.78±0.54 | 0.19±0.03 | 48.68±4.98 | ND | 1.94±0.76 | 83.15±11.13 | 8.99±0.24 | 4.54±0.12 |
| CS9 | ND | 1.42±0.09 | 2.01±0.47 | 0.35±0.02 | 58.69±2.45 | ND | 7.50±2.12 | 94.85±5.70 | 10.21±2.16 | 5.01±1.05 |
| CS10 | ND | 1.06±0.21 | 1.85±0.56 | 0.46±0.05 | 58.57±5.17 | ND | 7.15±0.82 | 104.42±7.43 | 9.38±0.23 | 4.61±0.16 |
| CS11 | ND | 3.25±0.11 | 1.61±0.31 | 0.38±0.01 | 61.12±2.03 | ND | 5.95±0.79 | 113.91±4.74 | 14.18±1.38 | 6.75±0.63 |
| CS12 | ND | 13.82±1.58 | 1.50±0.18 | 0.23±0.01 | 44.37±2.74 | ND | 1.48±0.31 | 99.28±5.52 | 14.9±0.77 | 7.44±0.43 |
| CS13 | ND | 3.52±0.48 | 1.11±0.16 | 0.20±0.03 | 45.15±7.36 | ND | 1.89±0.52 | 93.15±7.07 | 10.69±1.25 | 5.38±0.71 |
| CS14 | ND | 11.84±2.3 | 1.62±0.09 | 0.21±0.03 | 42.97±3.61 | ND | 2.64±0.98 | 91.05±2.90 | 11.28±0.7 | 5.45±0.36 |
| CS15 | ND | 6.08±2.67 | 0.86±0.15 | 0.18±0.05 | 45.33±9.61 | ND | 0.50±0.07 | 99.87±6.48 | 9.69±2.56 | 4.56±1.28 |
| CS16 | ND | 10.21±5.85 | 1.16±0.15 | 0.20±0.02 | 51.32±5.82 | ND | 0.61±0.32 | 101.87±3.32 | 10.55±1.20 | 4.94±0.47 |
| CS17 | ND | 7.04±3.11 | 1.34±0.57 | 0.17±0.05 | 41.77±17.09 | ND | 2.03±1.83 | 95.28±5.60 | 9.18±2.89 | 4.45±1.42 |
| CS18 | ND | 12.22±1.90 | 1.32±0.37 | 0.22±0.03 | 71.36±9.78 | ND | 4.65±1.46 | 92.89±2.22 | 9.65±0.94 | 4.62±0.39 |
| CS19 | ND | 1.55±0.33 | 1.66±0.13 | 0.33±0.04 | 65.20±7.82 | ND | 6.58±2.14 | 99.29±2.25 | 10.58±2.37 | 4.98±1.02 |
| CS20 | ND | 1.28±0.38 | 1.58±0.38 | 0.35±0.07 | 61.47±11.54 | ND | 7.96±0.21 | 101.19±8.55 | 11.08±1.20 | 5.22±0.43 |
| CS21 | ND | 1.49±0.48 | 1.17±0.07 | 0.29±0.07 | 61.66±10.83 | ND | 7.06±0.49 | 89.49±1.30 | 10.17±1.49 | 5.01±0.74 |

(*Continued next page*)

(*Table S5., continued*)

| Compound^a^ | Quinic acid | Fructose | Glucose | Mannitol | Inositol | Tryptophan | Sucrose | Trehalose |
| --- | --- | --- | --- | --- | --- | --- | --- | --- |
| KS1 | ND | 5.32±1.43 | 90.95±8.28 | 14.28±1.37 | 70.02±4.16 | 1.81±0.07 | 94.72±2.95 | ND |
| KS2 | ND | 7.08±2.37 | 131.54±44.85 | 14.85±2.96 | 52.10±5.09 | 1.65±0.19 | 93.42±7.59 | ND |
| KS3 | ND | 3.24±0.84 | 54.18±11.33 | 54.37±20.83 | 68.21±1.57 | 3.26±0.08 | 103.28±2.58 | ND |
| KS4 | ND | 5.08±0.71 | 71.41±8.05 | 16.20±2.57 | 59.89±4.01 | 1.90±0.11 | 96.98±5.45 | ND |
| KS5 | ND | 8.11±1.30 | 164.45±22.17 | 19.05±0.82 | 63.20±0.77 | 2.64±0.07 | 100.14±2.22 | ND |
| CS1 | ND | 9.71±2.22 | 99.01±19.75 | 27.08±6.52 | 73.59±0.75 | 2.66±0.15 | 97.83±1.82 | ND |
| CS2 | ND | 31.02±26.58 | 111.55±10.16 | 38.24±5.69 | 75.98±2.07 | 2.71±0.28 | 95.93±6.61 | ND |
| CS3 | ND | 38.74±13.73 | 182.32±41.77 | 24.24±7.22 | 77.03±3.22 | 2.89±0.18 | 101.81±1.37 | ND |
| CS4 | ND | 34.41±3.04 | 160.02±27.9 | 24.26±1.83 | 90.57±6.03 | 2.23±0.10 | 92.69±3.98 | ND |
| CS5 | ND | 7.60±1.46 | 72.28±12.55 | 12.46±0.46 | 81.93±3.78 | 1.81±0.17 | 90.27±4.48 | ND |
| CS6 | ND | 13.54±0.97 | 101.45±11.28 | 10.00±0.36 | 87.16±3.88 | 1.99±0.19 | 89.30±4.27 | ND |
| CS7 | ND | 12.34±6.43 | 94.78±25.87 | 11.43±0.32 | 82.02±3.21 | 1.52±0.16 | 85.94±3.05 | ND |
| CS8 | ND | 21.33±16.72 | 84.02±13.08 | 11.46±0.55 | 82.86±2.19 | 1.69±0.10 | 85.09±6.10 | ND |
| CS9 | ND | 9.30±1.57 | 91.70±8.29 | 11.39±1.62 | 67.80±6.89 | 2.03±0.10 | 97.57±9.30 | ND |
| CS10 | ND | 9.61±0.76 | 67.26±12.31 | 8.77±0.87 | 76.23±4.25 | 1.85±0.19 | 102.32±1.97 | ND |
| CS11 | ND | 8.66±4.18 | 80.93±28.16 | 20.91±10.86 | 82.50±4.75 | 2.16±0.19 | 109.1±8.34 | ND |
| CS12 | ND | 68.53±20.6 | 157.83±11.45 | 25.72±1.79 | 85.33±5.50 | 1.90±0.35 | 97.06±6.70 | ND |
| CS13 | ND | 7.17±0.80 | 66.57±22.67 | 12.14±0.94 | 79.30±1.89 | 1.41±0.14 | 95.16±1.29 | ND |
| CS14 | ND | 53.87±29.94 | 111.11±41.24 | 24.80±2.66 | 82.43±2.48 | 1.60±0.27 | 89.36±2.63 | ND |
| CS15 | ND | 6.14±2.04 | 78.73±16.77 | 62.00±25.00 | 86.80±4.73 | 1.06±0.19 | 90.86±6.00 | ND |
| CS16 | ND | 31.67±36.63 | 33.89±25.83 | 123.00±100.83 | 63.81±31.69 | 14.86±9.63 | 92.23±5.37 | ND |
| CS17 | ND | 8.71±3.98 | 66.47±45.61 | 55.99±31.43 | 60.45±24.71 | 4.22±4.81 | 85.59±5.58 | ND |
| CS18 | ND | 10.67±1.39 | 5.41±0.77 | 81.80±10.71 | 21.48±4.10 | 10.47±1.73 | 90.69±6.53 | ND |
| CS19 | ND | 10.29±2.22 | 104.54±29.83 | 13.81±2.15 | 73.66±5.77 | 2.24±0.03 | 93.08±5.35 | ND |
| CS20 | ND | 6.74±1.92 | 68.4±7.23 | 14.56±4.87 | 67.79±6.35 | 1.85±0.28 | 94.60±6.09 | ND |
| CS21 | ND | 6.54±1.96 | 72.06±23.61 | 14.93±1.24 | 68.67±1.85 | 1.85±0.27 | 92.08±6.33 | ND |

^a^ Each value is the mean of three replications ± standard deviation. ^b^ ND, Not detected.

Table S6. Composition and content (µg/g) of policosanol compounds in perilla (*Perilla frutescens*) cultivars

| Compound^a^ | C20-ol | C21-ol | C22-ol | C23-ol | C24-ol | C26-ol | C27-ol | C28-ol | C30-ol |
| --- | --- | --- | --- | --- | --- | --- | --- | --- | --- |
| KP1 | 9.28±0.76 | 1.80±0.25 | 16.71±0.29 | 0.86±0.53 | 22.01±6.80 | 32.22±10.10 | 10.81±1.18 | 145.84±40.28 | 63.41±17.76 |
| KP2 | 7.56±0.39 | 1.93±0.07 | 13.34±1.18 | 0.44±0.20 | 15.89±2.14 | 29.06±6.39 | 9.54±2.45 | 138.70±53.11 | 38.65±21.76 |
| KP3 | 6.22±0.44 | 1.82±0.13 | 14.21±1.40 | 0.65±0.13 | 18.82±0.77 | 25.15±1.48 | 9.42±1.30 | 107.84±24.93 | 41.22±19.17 |
| KP4 | 7.56±0.62 | 1.93±0.22 | 13.34±0.94 | 0.44±0.57 | 15.89±4.83 | 29.06±4.26 | 9.54±0.47 | 138.70±10.55 | 38.65±16.13 |
| CP1 | 7.59±1.17 | 1.67±0.17 | 13.96±2.12 | 1.81±0.28 | 25.11±2.58 | 35.23±13.57 | 8.75±0.72 | 90.83±39.14 | 62.20±19.80 |
| CP2 | 14.01±0.41 | 2.20±0.21 | 23.57±3.86 | 0.89±0.30 | 22.72±3.55 | 37.38±19.90 | 9.94±0.30 | 172.04±71.50 | 68.34±16.41 |
| CP3 | 9.78±0.74 | 1.78±0.04 | 27.12±1.89 | 1.98±0.12 | 34.63±2.60 | 47.53±6.06 | 11.36±0.78 | 169.53±50.58 | 58.67±4.94 |
| CP4 | 14.48±1.91 | 2.13±0.08 | 31.57±2.98 | 0.54±0.29 | 28.87±3.83 | 33.16±8.98 | 8.93±2.79 | 200.74±51.39 | 98.70±29.03 |
| CP5 | 7.73±0.28 | 1.64±0.08 | 23.03±2.20 | 2.56±0.41 | 37.22±0.91 | 43.97±7.83 | 11.50±0.23 | 129.25±27.77 | 56.45±21.59 |
| CP6 | 11.46±1.74 | 1.95±0.03 | 27.80±4.05 | 3.11±0.26 | 34.45±4.14 | 45.10±10.07 | 14.65±0.64 | 212.73±95.64 | 89.88±17.05 |
| CP7 | 9.41±0.75 | 1.89±0.26 | 21.09±1.02 | 1.12±0.64 | 28.48±2.52 | 43.75±11.41 | 11.10±1.19 | 167.93±78.00 | 70.81±24.59 |
| CP8 | 10.35±0.95 | 1.86±0.16 | 17.86±1.65 | 0.47±0.06 | 19.11±2.95 | 54.36±8.03 | 10.40±1.07 | 126.10±49.70 | 46.69±2.15 |
| CP9 | 16.65±1.70 | 2.11±0.20 | 28.89±4.27 | 0.91±0.47 | 24.16±7.41 | 43.28±19.43 | 10.58±2.25 | 114.81±3.41 | 47.92±12.54 |
| CP10 | 10.82±1.00 | 1.74±0.10 | 22.43±2.19 | 0.54±0.44 | 22.31±2.69 | 51.44±20.20 | 10.09±3.64 | 174.98±81.23 | 74.95±27.43 |
| CP11 | 10.96±0.28 | 1.74±0.13 | 22.30±2.38 | 0.68±0.28 | 20.71±2.34 | 56.66±18.14 | 11.11±1.65 | 275.66±38.04 | 57.51±25.67 |
| CP12 | 10.03±1.40 | 1.72±0.11 | 21.39±1.91 | 1.04±0.78 | 23.29±0.97 | 56.56±18.91 | 9.21±1.53 | 241.29±73.21 | 77.57±31.82 |
| CP13 | 10.21±1.48 | 1.87±0.19 | 21.53±1.65 | 0.54±0.39 | 24.91±2.81 | 43.14±3.34 | 11.68±0.60 | 236.31±18.65 | 58.20±24.42 |
| CP14 | 9.51±0.58 | 1.83±0.20 | 21.30±1.12 | 0.66±0.27 | 20.39±6.44 | 56.11±10.01 | 11.75±2.61 | 312.13±35.62 | 91.47±23.19 |
| CP15 | 9.58±1.37 | 1.84±0.25 | 22.27±4.57 | 0.75±0.52 | 22.49±5.49 | 31.07±4.53 | 11.04±2.15 | 211.42±71.45 | 57.74±5.30 |

^a^ Each value is the mean of three replications ± standard deviation.

C20, eicosanol; C21, heneicosanol; C22, docosanol; C23, tricosanol; C24, tetracosanol; C26, hexacosanol; C27, heptacosanol; C28, octacosanol; C30, triacontanol

| Compound^a^ | C20-ol | C21-ol | C22-ol | C23-ol | C24-ol | C26-ol | C27-ol | C28-ol | C30-ol |
| --- | --- | --- | --- | --- | --- | --- | --- | --- | --- |
| KS1 | 10.40±0.16 | 4.46±0.44 | 7.17±0.06 | ND^b^ | 15.21±0.05 | 11.25±0.03 | 15.22±0.06 | 40.63±0.17 | 8.92±0.74 |
| KS2 | 11.12±0.32 | 7.27±0.60 | 18.55±0.24 | ND | 13.06±0.32 | 8.27±0.20 | 11.07±0.78 | 28.24±1.17 | 6.70±0.24 |
| KS3 | 10.05±0.38 | 5.02±0.05 | 6.88±0.34 | ND | 14.40±0.48 | 12.29±0.26 | 16.92±0.74 | 45.11±0.64 | 10.49±0.88 |
| KS4 | 10.2±0.07 | 5.42±0.26 | 6.79±0.17 | ND | 14.50±0.42 | 11.32±0.25 | 15.52±0.27 | 44.53±1.43 | 10.24±0.42 |
| KS5 | 9.86±0.53 | 3.96±0.46 | 6.83±0.17 | ND | 14.34±0.7 | 10.76±0.30 | 14.87±1.02 | 43.23±3.78 | 10.63±0.06 |
| CS1 | 10.68±0.70 | 6.70±1.47 | 7.30±0.24 | 0.18±0.25 | 15.62±0.85 | 12.77±0.79 | 16.10±0.59 | 46.17±1.80 | 12.13±3.17 |
| CS2 | 10.85±0.38 | 7.75±0.47 | 7.74±0.29 | 0.03±0.03 | 15.29±0.54 | 13.38±0.33 | 17.19±1.01 | 50.83±1.43 | 12.21±2.81 |
| CS3 | 10.77±0.47 | 4.96±0.10 | 7.20±0.34 | ND | 15.3±0.73 | 12.42±0.37 | 15.87±0.45 | 45.57±1.54 | 9.97±0.48 |
| CS4 | 10.15±0.20 | 4.99±0.04 | 6.87±0.13 | ND | 14.69±0.32 | 11.58±0.56 | 15.63±0.33 | 41.68±1.03 | 8.75±0.82 |
| CS5 | 10.84±0.61 | 4.91±0.66 | 7.29±0.44 | ND | 15.37±0.77 | 12.11±0.59 | 16.10±1.98 | 47.37±5.23 | 9.72±1.23 |
| CS6 | 10.86±0.57 | 8.62±0.75 | 7.31±0.38 | 0.57±0.20 | 15.63±0.72 | 13.05±0.30 | 17.06±0.26 | 46.88±0.68 | 10.61±0.66 |
| CS7 | 9.97±0.38 | 6.07±0.59 | 6.54±0.18 | 0.01±0.01 | 14.42±0.59 | 11.86±0.64 | 15.75±1.45 | 44.75±2.92 | 9.74±0.20 |
| CS8 | 10.48±0.31 | 5.63±0.25 | 6.92±0.18 | ND | 14.58±0.83 | 11.70±0.23 | 16.19±0.53 | 45.62±3.53 | 9.55±1.71 |
| CS9 | 10.81±0.22 | 5.09±0.12 | 7.24±0.29 | ND | 15.37±0.58 | 12.18±0.47 | 15.61±0.09 | 46.69±3.35 | 10.21±0.98 |
| CS10 | 10.62±0.29 | 10.27±0.55 | 7.11±0.10 | 4.37±0.20 | 15.93±0.59 | 18.39±0.65 | 27.02±1.69 | 50.15±2.24 | 10.70±0.79 |
| CS11 | 10.48±0.03 | 9.93±0.68 | 6.99±0.10 | 4.04±1.02 | 16.29±0.13 | 18.01±1.00 | 27.60±1.21 | 41.45±2.06 | 9.38±0.72 |
| CS12 | 10.50±0.18 | 5.85±0.74 | 7.21±0.20 | ND | 15.19±0.45 | 12.11±0.26 | 17.21±0.79 | 48.59±1.23 | 14.04±1.99 |
| CS13 | 10.00±0.50 | 4.59±0.43 | 6.77±0.33 | ND | 14.39±0.58 | 11.47±0.48 | 15.69±1.05 | 44.34±3.38 | 10.27±0.74 |
| CS14 | 10.71±0.34 | 5.06±0.56 | 7.27±0.28 | ND | 15.65±0.63 | 12.68±0.47 | 16.20±1.05 | 46.20±1.58 | 12.35±2.23 |
| CS15 | 10.37±0.57 | 5.48±1.12 | 7.14±0.27 | ND | 14.99±0.55 | 10.29±0.22 | 12.43±0.77 | 36.80±1.31 | 9.36±0.17 |
| CS16 | 10.87±0.19 | 5.89±0.58 | 7.58±0.65 | ND | 15.52±0.77 | 10.50±0.52 | 12.75±0.82 | 39.18±3.32 | 9.47±0.24 |
| CS17 | 10.20±0.29 | 4.79±0.48 | 6.95±0.22 | ND | 14.72±0.43 | 12.05±0.51 | 15.66±0.51 | 48.08±0.53 | 9.81±0.19 |
| CS18 | 11.09±0.18 | 4.87±0.27 | 7.42±0.09 | ND | 15.72±0.09 | 12.18±0.23 | 16.33±1.09 | 48.80±2.29 | 11.38±0.52 |
| CS19 | 10.49±0.47 | 4.82±0.39 | 7.30±0.47 | ND | 15.11±0.7 | 12.98±0.79 | 16.50±1.58 | 50.08±3.75 | 11.02±0.67 |
| CS20 | 10.95±0.45 | 5.30±0.48 | 7.72±0.25 | ND | 15.18±0.73 | 12.95±0.58 | 17.65±1.05 | 49.58±1.07 | 10.73±0.41 |
| CS21 | 10.61±0.43 | 4.89±0.47 | 7.15±0.11 | ND | 15.35±0.37 | 12.19±0.78 | 16.50±0.29 | 51.31±2.56 | 12.24±2.23 |

Table S7. Composition and content (µg/g) of policosanol compounds in sesame (*Sesamum indicum*) cultivars

^a^ Each value is the mean of three replications ± standard deviation. ^b^ Not detected, ND

C20, eicosanol; C21, heneicosanol; C22, docosanol; C23, tricosanol; C24, tetracosanol; C26, hexacosanol; C27, heptacosanol; C28, octacosanol; C30, triacontanol

Table S8. Composition and content (µg/g) of sterol and terpenoid compounds in perilla (*Perilla frutescens*) cultivars

| Compound^a^ | β-Tocopherol | γ-Tocopherol | α-Tocopherol | Campesterol | Stigmasterol | β-Sitosterol | β-Amyrin | α-Amyrin |
| --- | --- | --- | --- | --- | --- | --- | --- | --- |
| KP1 | 5.39±0.42 | 121.51±15.15 | 15.17±1.30 | 250.43±18.20 | 56.26±17.94 | 840.62±73.69 | 8.22±1.22 | 20.36±1.19 |
| KP2 | 4.84±0.24 | 107.88±19.77 | 12.32±3.38 | 231.19±34.16 | 75.32±3.85 | 730.61±99.34 | 7.11±0.43 | 18.02±1.79 |
| KP3 | 4.96±0.35 | 103.35±17.10 | 11.79±1.80 | 232.32±34.15 | 68.52±10.74 | 724.88±95.40 | 7.38±0.50 | 18.81±2.32 |
| KP4 | 4.84±0.27 | 107.88±0.30 | 12.32±0.62 | 231.19±3.63 | 75.32±7.08 | 730.61±64.09 | 7.11±0.41 | 18.02±1.81 |
| CP1 | 4.80±0.27 | 58.85±4.07 | 7.24±1.05 | 157.07±7.61 | 34.74±3.15 | 413.09±28.12 | 5.64±0.57 | 12.06±1.02 |
| CP2 | 5.66±0.44 | 112.93±13.43 | 11.30±1.71 | 245.28±38.73 | 72.27±13.30 | 809.92±65.20 | 7.35±0.79 | 17.57±3.64 |
| CP3 | 5.39±0.61 | 81.22±16.74 | 9.04±1.47 | 271.03±30.01 | 65.67±13.33 | 645.11±73.20 | 4.20±0.35 | 4.67±0.39 |
| CP4 | 5.91±0.09 | 104.05±9.39 | 10.27±1.11 | 262.78±16.38 | 75.71±6.44 | 803.79±24.76 | 7.64±0.23 | 17.55±2.01 |
| CP5 | 4.58±0.63 | 63.98±8.49 | 6.36±0.36 | 177.49±7.85 | 38.59±3.33 | 436.69±14.49 | 4.51±0.66 | 4.16±0.55 |
| CP6 | 5.04±0.40 | 58.03±5.08 | 7.00±0.81 | 275.53±39.45 | 78.37±3.05 | 549.24±16.00 | 4.41±0.05 | 4.91±0.06 |
| CP7 | 5.35±0.50 | 68.01±6.42 | 9.83±1.46 | 243.60±3.62 | 54.66±1.94 | 627.31±48.64 | 4.29±0.32 | 4.77±0.35 |
| CP8 | 4.65±0.55 | 72.49±8.35 | 10.08±1.54 | 189.29±14.91 | 50.42±12.55 | 609.21±81.95 | 6.71±0.80 | 16.64±0.68 |
| CP9 | 4.70±0.15 | 107.73±14.17 | 8.05±1.04 | 239.69±22.02 | 62.17±13.25 | 774.99±105.83 | 6.77±0.37 | 18.28±2.09 |
| CP10 | 4.32±0.26 | 100.31±1.30 | 10.47±1.35 | 244.46±8.78 | 66.26±3.86 | 747.65±44.16 | 6.61±0.36 | 16.81±1.72 |
| CP11 | 4.18±0.18 | 87.24±16.49 | 10.00±1.85 | 232.37±17.28 | 57.20±6.77 | 719.10±56.58 | 6.38±0.34 | 16.35±0.51 |
| CP12 | 4.57±0.42 | 81.32±21.07 | 9.44±2.09 | 218.03±33.63 | 64.68±5.92 | 720.99±61.63 | 5.99±0.47 | 15.10±2.25 |
| CP13 | 4.50±0.21 | 83.87±16.32 | 8.90±1.53 | 189.43±11.09 | 51.28±5.66 | 623.19±47.42 | 6.10±0.36 | 13.71±2.95 |
| CP14 | 4.35±0.30 | 95.05±12.87 | 9.18±1.49 | 216.01±9.79 | 55.85±1.43 | 720.39±31.14 | 6.75±0.23 | 13.89±1.30 |
| CP15 | 4.39±0.31 | 101.73±24.50 | 8.58±1.96 | 223.10±41.61 | 61.65±4.25 | 727.19±66.22 | 5.88±0.38 | 14.26±2.54 |

^a^ Each value is the mean of three replications ± standard deviation.

Table S9. Composition and content (µg/g) of sterol and terpenoid compounds in sesame (*Sesamum indicum*) cultivars

| Compound^a^ | β-Tocopherol | γ-Tocopherol | α-Tocopherol | Campesterol | Stigmasterol | β-Sitosterol | β-Amyrin | α-Amyrin |
| --- | --- | --- | --- | --- | --- | --- | --- | --- |
| KS1 | ND^b^ | 66.79±2.34 | ND | 815.8±41.37 | 271.05±8.03 | 1232.76±27.18 | ND | ND |
| KS2 | ND | 65.48±3.09 | ND | 555.17±42.90 | 178.44±2.41 | 956.11±41.55 | ND | ND |
| KS3 | ND | 62.25±4.49 | ND | 570.71±66.36 | 223.43±13.7 | 1071.70±98.95 | ND | ND |
| KS4 | ND | 78.77±3.02 | ND | 1087.26±52.69 | 324.66±10.07 | 1454.11±31.19 | ND | ND |
| KS5 | ND | 72.07±10.78 | ND | 771.52±119.59 | 270.88±32.41 | 1227.91±217.29 | ND | ND |
| CS1 | ND | 99.47±15.91 | ND | 987.04±63.22 | 306.41±7.09 | 1237.38±54.59 | ND | ND |
| CS2 | ND | 106.09±2.14 | ND | 1005.15±37.44 | 335.38±13.33 | 1384.51±35.22 | ND | ND |
| CS3 | ND | 84.77±10.72 | ND | 736.71±58.07 | 263.19±14.3 | 1024.26±93.94 | ND | ND |
| CS4 | ND | 95.04±4.14 | ND | 866.50±46.20 | 286.53±14.18 | 1117.41±41.41 | ND | ND |
| CS5 | ND | 74.57±12.88 | ND | 735.52±120.75 | 224.76±28.72 | 923.83±139.00 | ND | ND |
| CS6 | ND | 101.91±19.05 | ND | 987.89±179.96 | 296.96±11.51 | 1290.97±179.97 | ND | ND |
| CS7 | ND | 86.25±5.69 | ND | 800.16±57.07 | 268.38±21.64 | 1102.68±35.55 | ND | ND |
| CS8 | ND | 82.13±1.74 | ND | 909.03±11.07 | 284.33±6.16 | 1169.47±37.28 | ND | ND |
| CS9 | ND | 81.37±10.09 | ND | 837.42±138.92 | 282.41±36.42 | 1053.68±125.05 | ND | ND |
| CS10 | ND | 142.88±11.32 | ND | 939.83±83.61 | 306.93±30.33 | 1247.92±81.12 | ND | ND |
| CS11 | ND | 129.32±11.49 | ND | 860.69±105.80 | 262.75±5.39 | 1147.43±119.22 | ND | ND |
| CS12 | ND | 78.32±4.89 | ND | 803.01±110.16 | 283.34±32.89 | 1130.41±119.8 | ND | ND |
| CS13 | ND | 80.54±2.68 | ND | 768.82±91.51 | 265.49±27.78 | 1050.33±70.17 | ND | ND |
| CS14 | ND | 73.26±10.8 | ND | 720.57±89.77 | 263.94±32.46 | 1003.59±114.48 | ND | ND |
| CS15 | ND | 60.12±6.64 | ND | 732.8±78.88 | 239.68±13.83 | 807.09±78.97 | ND | ND |
| CS16 | ND | 70.49±3.47 | ND | 1016.90±68.34 | 326.06±37.02 | 1214.14±59.40 | ND | ND |
| CS17 | ND | 63.96±10.28 | ND | 715.17±141.65 | 227.84±37.27 | 895.19±159.05 | ND | ND |
| CS18 | ND | 81.63±3.28 | ND | 921.06±55.38 | 280.78±19.58 | 1218.52±104.68 | ND | ND |
| CS19 | ND | 71.01±7.40 | ND | 746.30±69.17 | 260.18±27.95 | 971.33±123.82 | ND | ND |
| CS20 | ND | 76.61±6.23 | ND | 938.02±99.46 | 307.53±12.83 | 1166.35±90.74 | ND | ND |
| CS21 | ND | 82.15±14.17 | ND | 758.27±138.32 | 261.27±37.34 | 992.83±174.76 | ND | ND |

^a^ Each value is the mean of three replications ± standard deviation. ^b^ Not detected, ND

Table S10. Composition and content (mg/g) of fatty acids in perilla (*Perilla frutescens*) cultivars

| Compound^a^ | C12:0 | C14:0 | C16:1n7 | C16:0 | C18:2n6 | C18:3n3 | C18:1n9 | C18:0 | C20:0 | C22:0 | C24:0 |
| --- | --- | --- | --- | --- | --- | --- | --- | --- | --- | --- | --- |
| KP1 | 0.01±0.01 | 0.02±0.01 | 0.06±0.02 | 5.38±0.38 | 29.07±2.10 | 101.22±6.42 | 13.92±0.80 | 2.56±0.18 | 0.15±0.02 | 0.02±0.01 | 0.02±0.01 |
| KP2 | 0.01±0.01 | 0.02±0.01 | 0.06±0.01 | 4.82±0.10 | 33.72±1.11 | 95.88±3.19 | 15.29±0.46 | 2.70±0.03 | 0.17±0.01 | 0.02±0.01 | 0.02±0.01 |
| KP3 | 0.01±0.01 | 0.02±0.01 | 0.10±0.03 | 6.32±0.72 | 38.04±3.92 | 113.63±16.43 | 12.26±2.07 | 2.39±0.13 | 0.16±0.01 | 0.02±0.01 | 0.02±0.01 |
| KP4 | 0.01±0.01 | 0.02±0.01 | 0.09±0.02 | 5.78±0.28 | 37.95±1.84 | 99.24±7.06 | 12.04±0.58 | 2.24±0.09 | 0.15±0.01 | 0.03±0.01 | 0.02±0.01 |
| CP1 | 0.01±0.01 | 0.02±0.01 | 0.06±0.01 | 3.69±0.05 | 17.43±0.61 | 73.46±2.54 | 14.41±0.29 | 1.82±0.05 | 0.12±0.01 | 0.02±0.01 | 0.02±0.01 |
| CP2 | 0.01±0.01 | 0.02±0.01 | 0.07±0.01 | 5.24±0.21 | 32.11±1.11 | 113.83±2.69 | 10.4±0.39 | 1.76±0.04 | 0.09±0.01 | 0.01±0.01 | 0.02±0.01 |
| CP3 | 0.01±0.01 | 0.02±0.01 | 0.07±0.01 | 4.77±0.40 | 27.53±1.82 | 89.98±3.16 | 15.73±0.50 | 2.79±0.17 | 0.18±0.02 | 0.03±0.01 | 0.02±0.01 |
| CP4 | 0.01±0.01 | 0.02±0.01 | 0.07±0.01 | 5.29±0.28 | 38.81±2.63 | 115.01±4.91 | 10.86±0.63 | 1.79±0.10 | 0.09±0.01 | 0.01±0.01 | 0.02±0.01 |
| CP5 | 0.01±0.01 | 0.02±0.01 | 0.06±0.02 | 3.75±0.57 | 20.73±3.56 | 81.78±7.76 | 11.67±1.00 | 1.56±0.21 | 0.10±0.03 | 0.02±0.01 | 0.02±0.01 |
| CP6 | 0.01±0.01 | 0.02±0.01 | 0.05±0.01 | 3.18±0.13 | 21.99±1.03 | 70.03±3.30 | 7.80±0.36 | 1.43±0.12 | 0.10±0.01 | 0.02±0.01 | 0.02±0.01 |
| CP7 | 0.01±0.01 | 0.02±0.01 | 0.07±0.01 | 4.62±0.29 | 23.79±1.24 | 82.78±4.22 | 17.56±1.17 | 2.79±0.20 | 0.18±0.02 | 0.02±0.01 | 0.02±0.01 |
| CP8 | 0.01±0.01 | 0.02±0.01 | 0.08±0.01 | 5.21±0.06 | 35.45±1.12 | 106.67±3.69 | 17.21±1.03 | 2.64±0.02 | 0.14±0.01 | 0.02±0.01 | 0.02±0.01 |
| CP9 | 0.01±0.01 | 0.02±0.01 | 0.06±0.01 | 4.95±0.45 | 37.11±3.22 | 113.81±10.21 | 10.93±1.05 | 1.67±0.16 | 0.09±0.01 | 0.02±0.01 | 0.02±0.01 |
| CP10 | 0.01±0.01 | 0.02±0.01 | 0.09±0.01 | 5.53±0.15 | 42.09±2.84 | 105.14±4.02 | 12.59±0.54 | 2.17±0.04 | 0.12±0.01 | 0.02±0.01 | 0.02±0.01 |
| CP11 | 0.01±0.01 | 0.02±0.01 | 0.07±0.01 | 4.63±0.49 | 35.25±3.77 | 94.66±8.40 | 16.19±1.31 | 2.49±0.30 | 0.13±0.03 | 0.02±0.01 | 0.02±0.01 |
| CP12 | 0.01±0.01 | 0.02±0.01 | 0.07±0.01 | 5.43±0.20 | 35.58±1.61 | 106.67±4.72 | 16.78±0.21 | 2.69±0.11 | 0.14±0.01 | 0.02±0.01 | 0.02±0.01 |
| CP13 | 0.01±0.01 | 0.02±0.01 | 0.10±0.02 | 5.53±0.17 | 42.45±2.45 | 110.08±4.99 | 12.03±0.72 | 2.14±0.08 | 0.12±0.01 | 0.02±0.01 | 0.02±0.01 |
| CP14 | 0.01±0.01 | 0.02±0.01 | 0.10±0.01 | 5.41±0.25 | 34.42±1.10 | 108.26±2.35 | 14.94±0.56 | 2.08±0.05 | 0.12±0.01 | 0.02±0.01 | 0.02±0.01 |
| CP15 | 0.01±0.01 | 0.02±0.01 | 0.12±0.01 | 5.88±0.45 | 41.48±4.23 | 117.09±14.47 | 12.56±0.86 | 2.08±0.16 | 0.12±0.01 | 0.02±0.01 | 0.02±0.01 |

^a^ Each value is the mean of three replications ± standard deviation.

C12:0, lauric acid; C14:0, myristic acid; C16:1n7, palmitoleic acid; C16:0, palmitic acid; C18:2n6, linoleic acid; C18:3n3, α-linolenic acid; C18:1n9, oleic acid; C18:0, stearic acid; C20:0, arachidic acid; C22:0, behenic acid; C24:0, lignoceric acid.

Table S11. Composition and content (mg/g) of fatty acids in sesame (*Sesamum indicum*) cultivars

| Compound^a^ | C12:0 | C14:0 | C16:1n7 | C16:0 | C18:2n6 | C18:3n3 | C18:1n9 | C18:0 | C20:0 | C22:0 | C24:0 |
| --- | --- | --- | --- | --- | --- | --- | --- | --- | --- | --- | --- |
| KS1 | 0.01±0.01 | 0.03±0.01 | 0.17±0.01 | 11.32±1.59 | 128.46±21.39 | ND^b^ | 63.65±8.48 | 8.76±1.07 | 1.04±0.16 | 0.17±0.03 | 0.11±0.02 |
| KS2 | 0.01±0.01 | 0.02±0.01 | 0.15±0.03 | 9.35±1.77 | 112.79±22.01 | ND | 58.48±11.21 | 7.95±1.36 | 0.88±0.13 | 0.15±0.02 | 0.11±0.02 |
| KS3 | 0.01±0.01 | 0.03±0.01 | 0.14±0.01 | 9.61±0.19 | 103.29±1.69 | ND | 61.28±1.94 | 7.34±0.18 | 0.90±0.03 | 0.15±0.01 | 0.12±0.01 |
| KS4 | 0.01±0.01 | 0.02±0.01 | 0.20±0.01 | 11.19±0.48 | 120.52±7.31 | ND | 60.10±5.60 | 7.67±0.48 | 1.03±0.06 | 0.19±0.02 | 0.13±0.02 |
| KS5 | 0.01±0.01 | 0.02±0.01 | 0.13±0.01 | 8.68±0.43 | 110.59±1.05 | ND | 60.04±1.06 | 7.73±0.34 | 0.98±0.05 | 0.18±0.01 | 0.10±0.01 |
| CS1 | 0.01±0.01 | 0.02±0.01 | 0.13±0.01 | 9.67±1.03 | 119.98±14.00 | ND | 57.19±7.47 | 6.90±0.83 | 0.72±0.06 | 0.10±0.01 | 0.06±0.01 |
| CS2 | 0.01±0.01 | 0.02±0.01 | 0.17±0.01 | 11.04±0.58 | 134.76±7.26 | ND | 61.91±2.87 | 8.19±0.34 | 1.02±0.03 | 0.18±0.01 | 0.11±0.01 |
| CS3 | 0.01±0.01 | 0.02±0.01 | 0.16±0.01 | 10.66±0.37 | 125.19±6.30 | ND | 55.55±2.36 | 7.54±0.29 | 0.95±0.03 | 0.17±0.01 | 0.10±0.01 |
| CS4 | 0.01±0.01 | 0.02±0.01 | 0.17±0.02 | 10.28±0.22 | 116.50±5.11 | ND | 53.64±2.30 | 7.55±0.08 | 1.01±0.02 | 0.19±0.01 | 0.12±0.01 |
| CS5 | 0.02±0.01 | 0.02±0.01 | 0.18±0.02 | 12.00±1.46 | 131.98±15.76 | ND | 63.57±5.96 | 8.85±0.85 | 1.05±0.08 | 0.19±0.01 | 0.10±0.01 |
| CS6 | 0.02±0.01 | 0.02±0.01 | 0.15±0.02 | 10.38±0.15 | 124.97±4.79 | ND | 58.85±1.94 | 8.47±0.29 | 1.04±0.05 | 0.20±0.02 | 0.11±0.02 |
| CS7 | 0.02±0.01 | 0.03±0.01 | 0.16±0.01 | 10.71±0.36 | 122.82±4.65 | ND | 57.81±1.41 | 8.39±0.17 | 1.03±0.03 | 0.19±0.02 | 0.11±0.02 |
| CS8 | 0.02±0.01 | 0.02±0.01 | 0.14±0.02 | 10.15±0.39 | 119.04±5.39 | ND | 53.96±2.99 | 7.78±0.47 | 0.97±0.06 | 0.19±0.01 | 0.11±0.02 |
| CS9 | 0.02±0.01 | 0.03±0.01 | 0.20±0.02 | 11.42±0.6 | 127.65±7.60 | ND | 57.37±2.95 | 8.04±0.39 | 1.02±0.05 | 0.20±0.01 | 0.11±0.01 |
| CS10 | 0.02±0.01 | 0.02±0.01 | 0.18±0.02 | 10.61±0.48 | 120.24±2.77 | ND | 53.57±1.53 | 7.50±0.32 | 0.93±0.07 | 0.17±0.02 | 0.10±0.02 |
| CS11 | 0.02±0.01 | 0.02±0.01 | 0.16±0.02 | 10.57±0.14 | 121.15±5.21 | ND | 54.91±1.60 | 7.59±0.23 | 0.92±0.02 | 0.17±0.01 | 0.09±0.02 |
| CS12 | 0.02±0.01 | 0.02±0.01 | 0.19±0.03 | 9.65±1.01 | 113.90±12.06 | ND | 50.66±5.32 | 6.86±0.84 | 0.86±0.13 | 0.16±0.03 | 0.10±0.01 |
| CS13 | 0.01±0.01 | 0.02±0.01 | 0.12±0.01 | 8.45±0.29 | 101.61±5.16 | ND | 46.99±2.12 | 6.31±0.20 | 0.77±0.01 | 0.14±0.01 | 0.08±0.01 |
| CS14 | 0.01±0.01 | 0.02±0.01 | 0.14±0.01 | 9.05±0.49 | 110.22±8.10 | ND | 48.43±2.55 | 6.67±0.34 | 0.83±0.03 | 0.15±0.01 | 0.09±0.01 |
| CS15 | 0.01±0.01 | 0.02±0.01 | 0.13±0.04 | 8.25±1.28 | 97.85±15.27 | ND | 42.90±6.32 | 5.94±0.92 | 0.80±0.15 | 0.16±0.04 | 0.09±0.02 |
| CS16 | 0.01±0.01 | 0.02±0.01 | 0.12±0.03 | 8.30±0.87 | 97.21±9.15 | ND | 42.18±4.56 | 6.10±0.60 | 0.81±0.09 | 0.17±0.02 | 0.09±0.01 |
| CS17 | 0.01±0.01 | 0.02±0.01 | 0.15±0.03 | 9.12±1.29 | 100.81±12.52 | ND | 46.28±5.79 | 6.67±0.97 | 0.84±0.13 | 0.15±0.03 | 0.09±0.02 |
| CS18 | 0.01±0.01 | 0.02±0.01 | 0.14±0.02 | 9.21±0.09 | 102.77±2.24 | ND | 50.18±2.13 | 6.96±0.30 | 0.87±0.04 | 0.15±0.01 | 0.09±0.01 |
| CS19 | 0.01±0.01 | 0.02±0.01 | 0.15±0.03 | 9.28±1.36 | 103.55±14.60 | ND | 47.19±5.75 | 6.50±0.95 | 0.83±0.13 | 0.15±0.03 | 0.09±0.02 |
| CS20 | 0.01±0.01 | 0.02±0.01 | 0.15±0.02 | 9.39±0.18 | 112.09±0.40 | ND | 49.51±0.98 | 7.00±0.04 | 0.86±0.01 | 0.15±0.01 | 0.09±0.01 |
| CS21 | 0.01±0.01 | 0.02±0.01 | 0.13±0.02 | 9.25±0.25 | 108.17±3.19 | ND | 49.86±1.56 | 6.57±0.14 | 0.83±0.01 | 0.15±0.01 | 0.09±0.01 |

^a^ Each value is the mean of three replications ± standard deviation. ^b^ Not detected, ND

Table S12. OPLS-DA loading and VIP values of variables of perilla (*Perilla frutescens*) cultivars.

| Metabolite name | OPLS 1 | OPLS 2 | VIP | Metabolite name | OPLS 1 | OPLS 2 | VIP |
| --- | --- | --- | --- | --- | --- | --- | --- |
| C20-ol | 0.14064 | -0.03600 | 1.01467 | Threonine | -0.11255 | 0.17402 | 1.10329 |
| C21-ol | 0.03261 | -0.01265 | 0.23887 | β-Alanine | 0.04391 | 0.25019 | 1.13293 |
| C22-ol | 0.17191 | 0.06163 | 1.25444 | Malic acid | -0.12150 | 0.09304 | 0.95606 |
| C23-ol | 0.10275 | 0.15765 | 1.00357 | Aspartic acid | 0.03309 | 0.10668 | 0.52074 |
| C24-ol | 0.11578 | 0.13665 | 1.01727 | Methionine | -0.17104 | 0.09412 | 1.28619 |
| C26-ol | 0.17085 | -0.02321 | 1.22207 | 4-Aminobutyric acid | 0.14946 | 0.06989 | 1.10796 |
| β-Tocopherol | -0.09862 | 0.06835 | 0.76329 | Threonic acid | 0.08724 | 0.16428 | 0.94753 |
| γ-Tocopherol | -0.17839 | -0.07856 | 1.31677 | Glutamic acid | 0.02481 | 0.10678 | 0.49723 |
| C27-ol | 0.06706 | 0.12166 | 0.71333 | Phenylalanine | 0.02312 | 0.13732 | 0.61994 |
| C28-ol | 0.09236 | -0.02237 | 0.66553 | Glutamine | -0.13691 | 0.16435 | 1.20998 |
| α-Tocopherol | -0.23649 | -0.04395 | 1.69656 | Citric acid | -0.11277 | 0.06804 | 0.85665 |
| Campesterol | -0.0863 | 0.11993 | 0.80673 | Isocitric acid | -0.11488 | 0.07307 | 0.87846 |
| C30-ol | 0.03277 | 0.03392 | 0.27633 | Quinic acid | -0.08785 | 0.12621 | 0.83294 |
| Stigmasterol | -0.10936 | 0.07304 | 0.84183 | Fructose | 0.08582 | 0.16195 | 0.93323 |
| β-Sitosterol | -0.15962 | -0.05993 | 1.16731 | Glucose | 0.08340 | 0.24061 | 1.20411 |
| β-Amyrin | -0.15456 | -0.17706 | 1.34448 | Mannitol | 0.03566 | -0.10402 | 0.51917 |
| α-Amyrin | -0.17131 | -0.17250 | 1.43346 | Inositol | 0.02428 | 0.21084 | 0.93379 |
| Lactic acid | 0.16052 | -0.00541 | 1.14448 | Tryptophan | 0.0085 | -0.14366 | 0.62815 |
| Glycolic acid | 0.24136 | 0.13648 | 1.82011 | Sucrose | 0.10898 | 0.02060 | 0.78199 |
| Alanine | 0.03188 | 0.13612 | 0.63449 | Trehalose | -0.13567 | 0.09993 | 1.06035 |
| Valine | 0.02580 | 0.20980 | 0.93138 | C12:0 | -0.07251 | -0.02172 | 0.52542 |
| Ethanolamine | 0.06704 | 0.05912 | 0.54274 | C14:0 | -0.17030 | -0.04960 | 1.23299 |
| Leucine | 0.04405 | 0.18240 | 0.85364 | C16:1n7 | -0.02835 | -0.07789 | 0.39464 |
| Glycerol | 0.08698 | -0.08742 | 0.72739 | C16:0 | -0.13997 | -0.12616 | 1.13879 |
| Phosphoric acid | 0.17103 | 0.15519 | 1.39372 | C18:2n6 | -0.05478 | -0.13831 | 0.71746 |
| Isoleucine | 0.06361 | 0.13324 | 0.73610 | C18:3n3 | -0.03879 | -0.16874 | 0.78469 |
| Nicotinic acid | 0.12966 | -0.06224 | 0.96311 | C18:1n9 | -0.05381 | -0.03436 | 0.41168 |
| Succinic acid | 0.12289 | 0.12146 | 1.02313 | C18:0 | -0.16462 | 0.00655 | 1.17376 |
| Glycine | 0.10759 | 0.16099 | 1.03876 | C20:0 | -0.19887 | 0.09418 | 1.47567 |
| Glyceric acid | 0.12386 | 0.22713 | 1.32537 | C22:0 | -0.17769 | 0.11012 | 1.35423 |
| Fumaric acid | 0.07564 | -0.04204 | 0.56935 | C24:0 | -0.06334 | 0.11923 | 0.68780 |
| Serine | 0.04798 | 0.23568 | 1.08121 |  |  |  |  |

C20-ol, eicosanol; C21-ol, heneicosanol; C22-ol, docosanol; C23-ol, tricosanol; C24-ol, tetracosanol; C26-ol, hexacosanol; C27-ol, heptacosanol; C28-ol, octacosanol; C30-ol, triacontanol; C12:0, lauric acid; C14:0, myristic acid; C16:1n7, palmitoleic acid; C16:0, palmitic acid; C18:2n6, linoleic acid; C18:3n3, α-linolenic acid; C18:1n9, oleic acid; C18:0, stearic acid; C20:0, arachidic acid; C22:0, behenic acid; C24:0, lignoceric acid.

Table S13. OPLS-DA loading and VIP values of variables of sesame (*Sesamum indicum*) cultivars.

| Metabolite name | OPLS 1 | OPLS 2 | VIP | Metabolite name | OPLS 1 | OPLS 2 | VIP |
| --- | --- | --- | --- | --- | --- | --- | --- |
| C20-ol | 0.07223 | -0.08409 | 0.59239 | Threonine | -0.09168 | -0.18751 | 0.98426 |
| C21-ol | 0.10494 | -0.09243 | 0.79526 | Malic acid | -0.10245 | -0.20072 | 1.07158 |
| C22-ol | -0.18205 | -0.02932 | 1.21716 | Aspartic acid | -0.05179 | -0.19601 | 0.87792 |
| C23-ol | 0.09575 | -0.0628 | 0.68757 | 4-Aminobutyric acid | 0.17399 | 0.06717 | 1.19018 |
| C24-ol | 0.21051 | -0.06032 | 1.42241 | Threonic acid | -0.17461 | -0.19417 | 1.41046 |
| C26-ol | 0.18130 | -0.09292 | 1.26545 | Glutamic acid | -0.07250 | -0.2653 | 1.19467 |
| γ-Tocopherol | 0.16299 | -0.12687 | 1.20378 | Phenylalanine | 0.16839 | -0.21254 | 1.42189 |
| C27-ol | 0.14625 | -0.07797 | 1.02465 | Xylose | -0.06349 | -0.22465 | 1.01730 |
| C28-ol | 0.17477 | -0.03948 | 1.17407 | Galactose | 0.04318 | -0.14450 | 0.66098 |
| Campesterol | 0.13019 | -0.02617 | 0.87281 | Citric acid | -0.11561 | -0.18356 | 1.07861 |
| C30-ol | 0.11406 | -0.02300 | 0.76476 | Isocitric acid | -0.12537 | -0.17449 | 1.10111 |
| Stigmasterol | 0.13660 | -0.03872 | 0.92268 | Fructose | 0.13085 | -0.0197 | 0.87429 |
| β-Sitosterol | -0.00310 | -0.05719 | 0.23648 | Glucose | -0.02043 | -0.13022 | 0.55338 |
| Lactic acid | -0.02014 | -0.10537 | 0.45431 | Mannitol | 0.04332 | 0.05883 | 0.37656 |
| Glycolic acid | -0.05727 | -0.18857 | 0.86522 | Inositol | 0.14223 | -0.02882 | 0.95371 |
| Alanine | 0.22345 | -0.0026 | 1.48668 | Tryptophan | 0.04027 | 0.03248 | 0.29945 |
| Valine | 0.01653 | -0.2415 | 1.00097 | Sucrose | -0.04416 | -0.15727 | 0.71141 |
| Leucine | -0.01976 | -0.22556 | 0.93849 | C12:0 | 0.09969 | 0.02579 | 0.67170 |
| Glycerol | 0.15013 | 0.11487 | 1.10526 | C14:0 | -0.15752 | -0.0165 | 1.05021 |
| Phosphoric acid | 0.15949 | -0.01175 | 1.06218 | C16:1n7 | -0.02979 | -0.12997 | 0.57095 |
| Isoleucine | -0.06703 | -0.22879 | 1.04269 | C16:0 | -0.01574 | -0.18847 | 0.78347 |
| Proline | -0.27063 | -0.07245 | 1.82508 | C18:2n6 | -0.00631 | -0.21556 | 0.88904 |
| Succinic acid | 0.1534 | -0.07605 | 1.06758 | C18:1n9 | -0.14789 | -0.19027 | 1.25796 |
| Glycine | 0.23374 | -0.04755 | 1.56738 | C18:0 | -0.09267 | -0.16981 | 0.93245 |
| Glyceric acid | 0.16402 | 0.00191 | 1.09123 | C20:0 | -0.06273 | -0.14005 | 0.71209 |
| Fumaric acid | 0.05047 | -0.00269 | 0.33591 | C22:0 | 0.01202 | -0.06678 | 0.28647 |
| Serine | 0.05206 | -0.23677 | 1.03507 | C24:0 | -0.13699 | -0.05705 | 0.94123 |

C20-ol, eicosanol; C21-ol, heneicosanol; C22-ol, docosanol; C23-ol, tricosanol; C24-ol, tetracosanol; C26-ol, hexacosanol; C27-ol, heptacosanol; C28-ol, octacosanol; C30-ol, triacontanol; C12:0, lauric acid; C14:0, myristic acid; C16:1n7, palmitoleic acid; C16:0, palmitic acid; C18:2n6, linoleic acid; C18:3n3, α-linolenic acid; C18:1n9, oleic acid; C18:0, stearic acid; C20:0, arachidic acid; C22:0, behenic acid; C24:0, lignoceric acid.
