# Supplementary material for: Metabolite Profiling and Chemometric Study for the Discrimination Analyses of Geographic Origin of Perilla (Perilla frutescens) and Sesame (Sesamum indicum) Seeds
Source: Foods. 2020 Jul 24;9(8):989. doi: 10.3390/foods9080989 (PMC7466206; doi:10.3390/foods9080989)
Supplement: Supplementary file 1 [file foods-09-00989-s001.zip › (Proof)PS_supplementary Figures_revision_Final_ver.docx]

Supplementary figures

Metabolite Profiling and Chemometric Study for the Discrimination Analyses of Geographic Origin of Perilla (*Perilla frutescens*) and Sesame (*Sesamum indicum*) Seeds

Tae Jin Kim ^1, †^, Jeong Gon Park ^1, †^, Hyun Young Kim ^2^, Sun-Hwa Ha ^3^, Bumkyu Lee ^4^, Sang Un Park ^5^, Woo Duck Seo ^2,^* and Jae Kwang Kim ^1,^*

^1^ Division of Life Sciences, College of Life Sciences and Bioengineering, Incheon National University., Incheon, 22012, Republic of Korea; [f91gd@inu.ac.kr](mailto:f91gd@inu.ac.kr) (T.J.K.); [parkjk1132@naver.com](mailto:parkjk1132@naver.com) (J.G.P.)

^2^ Division of Crop Foundation, National Institute of Crop Science, Rural Development Administration, Wanju, Jeonbuk, 55365, Republic of Korea; [hkkim84@korea.kr](mailto:hkkim84@korea.kr) (H.Y.K.)

^3^ Department of Genetic Engineering and Graduate School of Biotechnology, Kyung Hee University, Yongin 17104, Republic of Korea; [sunhwa@khu.ac.kr](mailto:sunhwa@khu.ac.kr) (S.-H.H.)

^4^  Department of Environment Science & Biotechnology, Jeonju University, Jeonju 55069, Republic of Korea; [leebk@jj.ac.kr](mailto:leebk@jj.ac.kr) (B.L.)

^5^  Department of Crop Science, Chungnam National University, 99 Daehak-ro, Yuseong-gu, Daejeon 34134, Republic of Korea; supark@cnu.ac.kr (S.U.P.)

***** Correspondence: [swd2002@korea.kr](mailto:swd2002@korea.kr) (W.D.S.); Tel.: +82-63-238-5305; [kjkpj@inu.ac.kr](mailto:kjkpj@inu.ac.kr) (J.K.K.); Tel.: +82-32-835-8241

**^†^** These authors contributed equally to this work

Supplementary figures

**Figure S1**. PCA score (A) and loading (B) plots of perilla (Perilla frutescens) seeds from Korea and China. C20-ol, eicosanol; C21-ol, heneicosanol; C22-ol, docosanol; C23-ol, tricosanol; C24-ol, tetracosanol; C26-ol, hexacosanol; C27-ol, heptacosanol; C28-ol, octacosanol; C30-ol, triacontanol; C12:0, lauric acid; C14:0, myristic acid; C16:1n7, palmitoleic acid; C16:0, palmitic acid; C18:2n6, linoleic acid; C18:3n3, α-linolenic acid; C18:1n9, oleic acid; C18:0, stearic acid; C20:0, arachidic acid; C22:0, behenic acid; C24:0, lignoceric acid.

**Figure S2**. PCA score (A) and loading (B) plots of sesame (Sesamum indicum) seeds from Korea and China. C20-ol, eicosanol; C21-ol, heneicosanol; C22-ol, docosanol; C23-ol, tricosanol; C24-ol, tetracosanol; C26-ol, hexacosanol; C27-ol, heptacosanol; C28-ol, octacosanol; C30-ol, triacontanol; C12:0, lauric acid; C14:0, myristic acid; C16:1n7, palmitoleic acid; C16:0, palmitic acid; C18:2n6, linoleic acid; C18:3n3, α-linolenic acid; C18:1n9, oleic acid; C18:0, stearic acid; C20:0, arachidic acid; C22:0, behenic acid; C24:0, lignoceric acid.

**Figure S3**. OPLS-DA score (A) and loading (B) plots of perilla (Perilla frutescens) seeds from Korea and China. C20-ol, eicosanol; C21-ol, heneicosanol; C22-ol, docosanol; C23-ol, tricosanol; C24-ol, tetracosanol; C26-ol, hexacosanol; C27-ol, heptacosanol; C28-ol, octacosanol; C30-ol, triacontanol; C12:0, lauric acid; C14:0, myristic acid; C16:1n7, palmitoleic acid; C16:0, palmitic acid; C18:2n6, linoleic acid; C18:3n3, α-linolenic acid; C18:1n9, oleic acid; C18:0, stearic acid; C20:0, arachidic acid; C22:0, behenic acid; C24:0, lignoceric acid.

**Figure S4**. OPLS-DA score (A) and loading (B) plots of sesame (Sesamum indicum) seeds from Korea and China. C20-ol, eicosanol; C21-ol, heneicosanol; C22-ol, docosanol; C23-ol, tricosanol; C24-ol, tetracosanol; C26-ol, hexacosanol; C27-ol, heptacosanol; C28-ol, octacosanol; C30-ol, triacontanol; C12:0, lauric acid; C14:0, myristic acid; C16:1n7, palmitoleic acid; C16:0, palmitic acid; C18:2n6, linoleic acid; C18:3n3, α-linolenic acid; C18:1n9, oleic acid; C18:0, stearic acid; C20:0, arachidic acid; C22:0, behenic acid; C24:0, lignoceric acid.

**Figure S5.** PCA score plots and Hostelling T^2^ range column plots of perilla (Perilla frutescens) seeds from Korea and China for outlier removal process.

**Figure S6.** PCA score plots and Hostelling T^2^ range column plots of sesame (Sesamum indicum) seeds from Korea and China for outlier removal process.

**Figure S7.** OPLS–DA score plots and VIP (variable importance in the projection) plots of perilla (A) and sesame (B) seeds from Korea and China outlier removal data sets. C20-ol, eicosanol; C21-ol, heneicosanol; C22-ol, docosanol; C23-ol, tricosanol; C24-ol, tetracosanol; C26-ol, hexacosanol; C27-ol, heptacosanol; C28-ol, octacosanol; C30-ol, triacontanol; C12:0, lauric acid; C14:0, myristic acid; C16:1n7, palmitoleic acid; C16:0, palmitic acid; C18:2n6, linoleic acid; C18:3n3, α-linolenic acid; C18:1n9, oleic acid; C18:0, stearic acid; C20:0, arachidic acid; C22:0, behenic acid; C24:0, lignoceric acid.


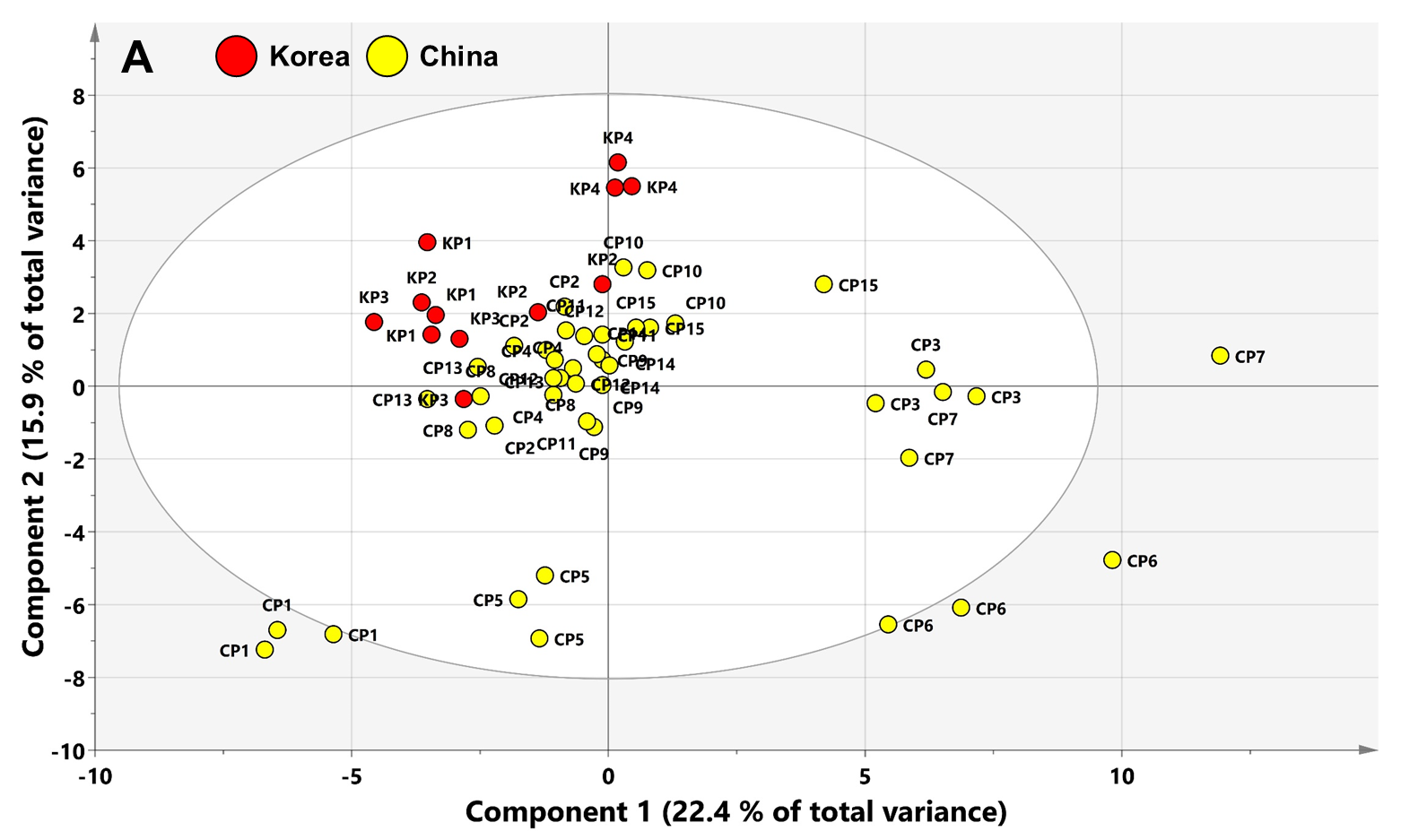
Figure S1. PCA score (A) and loading (B) plots of perilla (*Perilla frutescens*) seeds from Korea and China.
C20-ol, eicosanol; C21-ol, heneicosanol; C22-ol, docosanol; C23-ol, tricosanol; C24-ol, tetracosanol; C26-ol, hexacosanol; C27-ol, heptacosanol; C28-ol, octacosanol; C30-ol, triacontanol; C12:0, lauric acid; C14:0, myristic acid; C16:1n7, palmitoleic acid; C16:0, palmitic acid; C18:2n6, linoleic acid; C18:3n3, α-linolenic acid; C18:1n9, oleic acid; C18:0, stearic acid; C20:0, arachidic acid; C22:0, behenic acid; C24:0, lignoceric acid.

(*Continued next page*)

(*Figure S1., continued*)


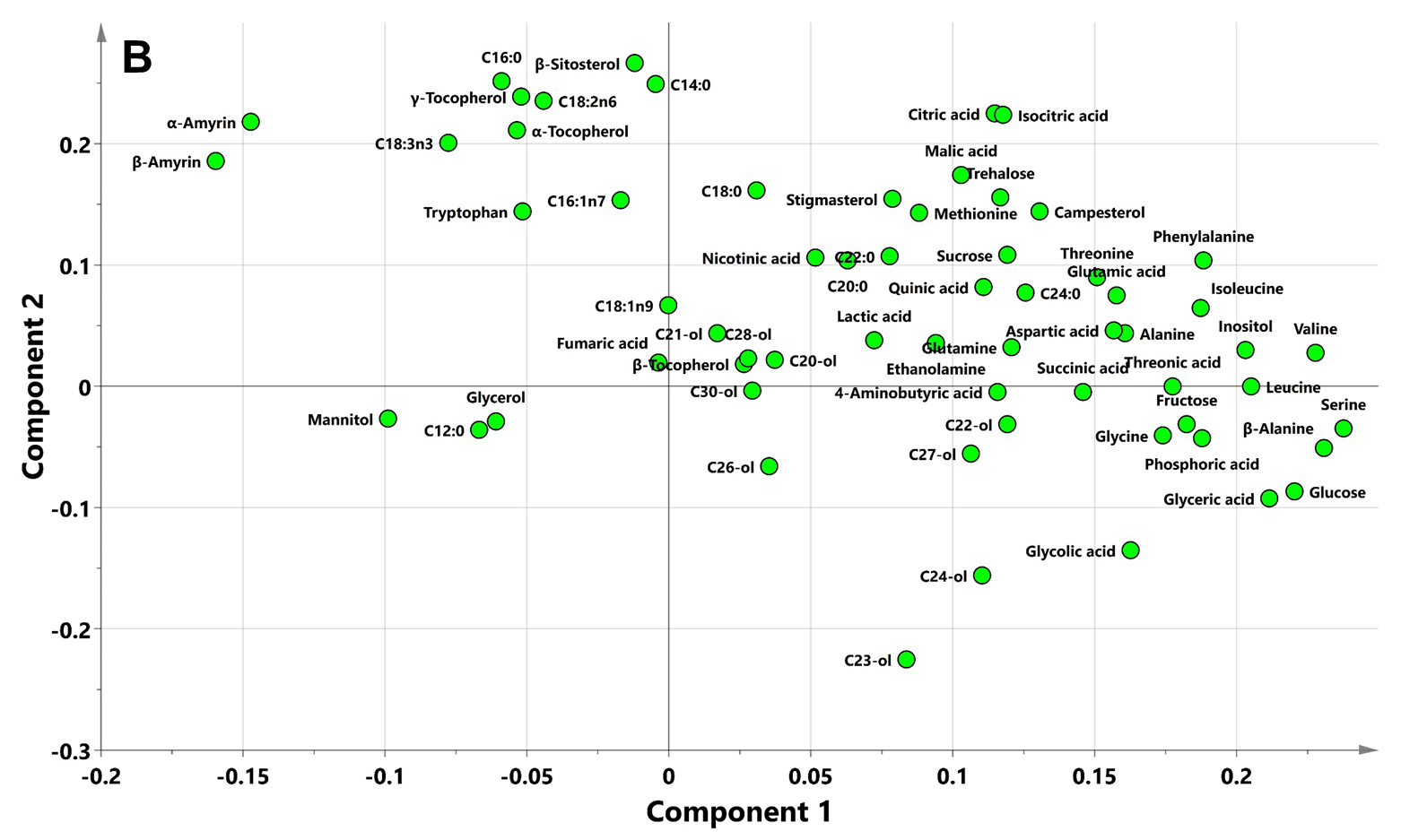

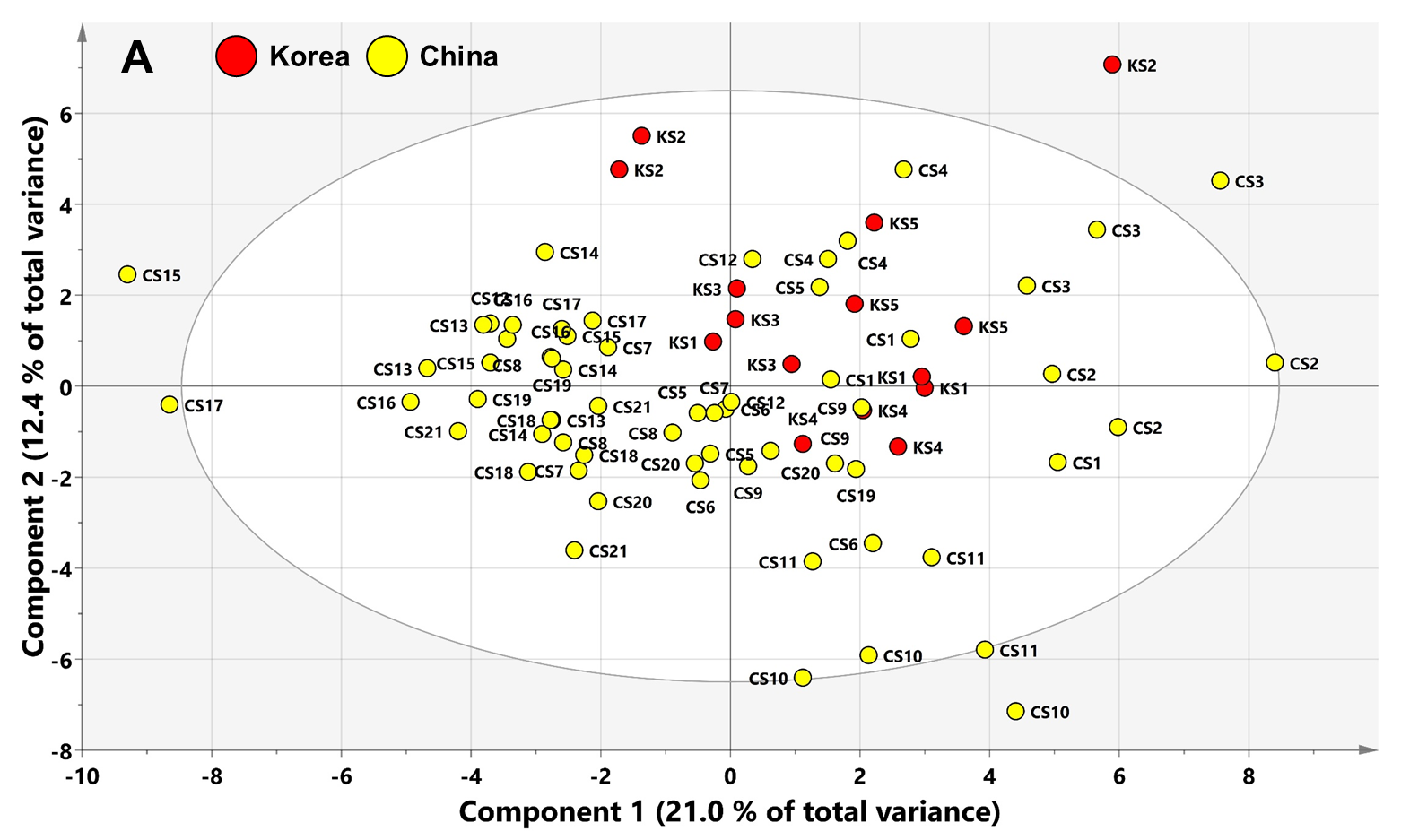
Figure S2. PCA score (A) and loading (B) plots of sesame (*Sesamum indicum*) seeds from Korea and China.
C20-ol, eicosanol; C21-ol, heneicosanol; C22-ol, docosanol; C23-ol, tricosanol; C24-ol, tetracosanol; C26-ol, hexacosanol; C27-ol, heptacosanol; C28-ol, octacosanol; C30-ol, triacontanol; C12:0, lauric acid; C14:0, myristic acid; C16:1n7, palmitoleic acid; C16:0, palmitic acid; C18:2n6, linoleic acid; C18:3n3, α-linolenic acid; C18:1n9, oleic acid; C18:0, stearic acid; C20:0, arachidic acid; C22:0, behenic acid; C24:0, lignoceric acid.

(*Continued next page*)


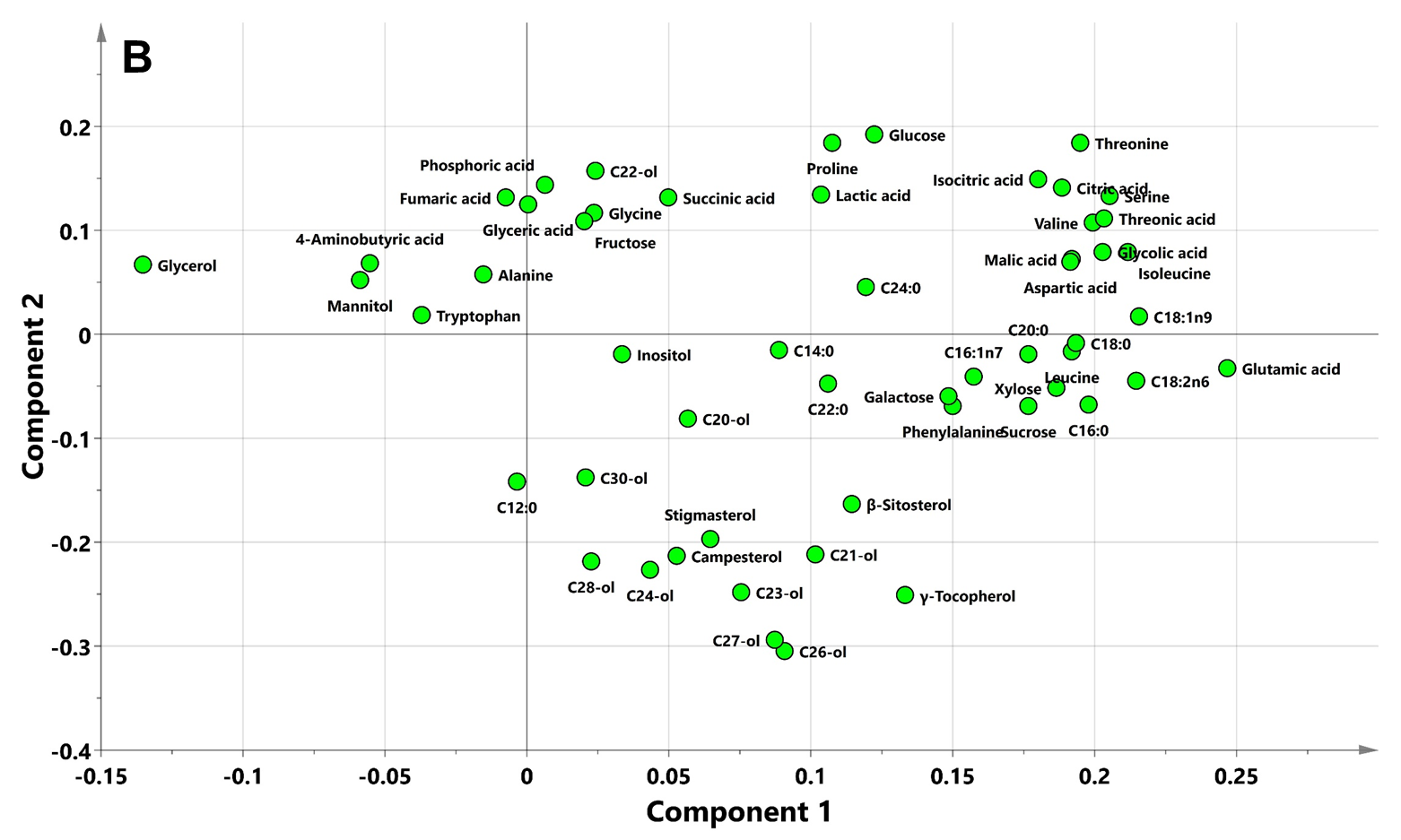
(*Figure S2., continued*)


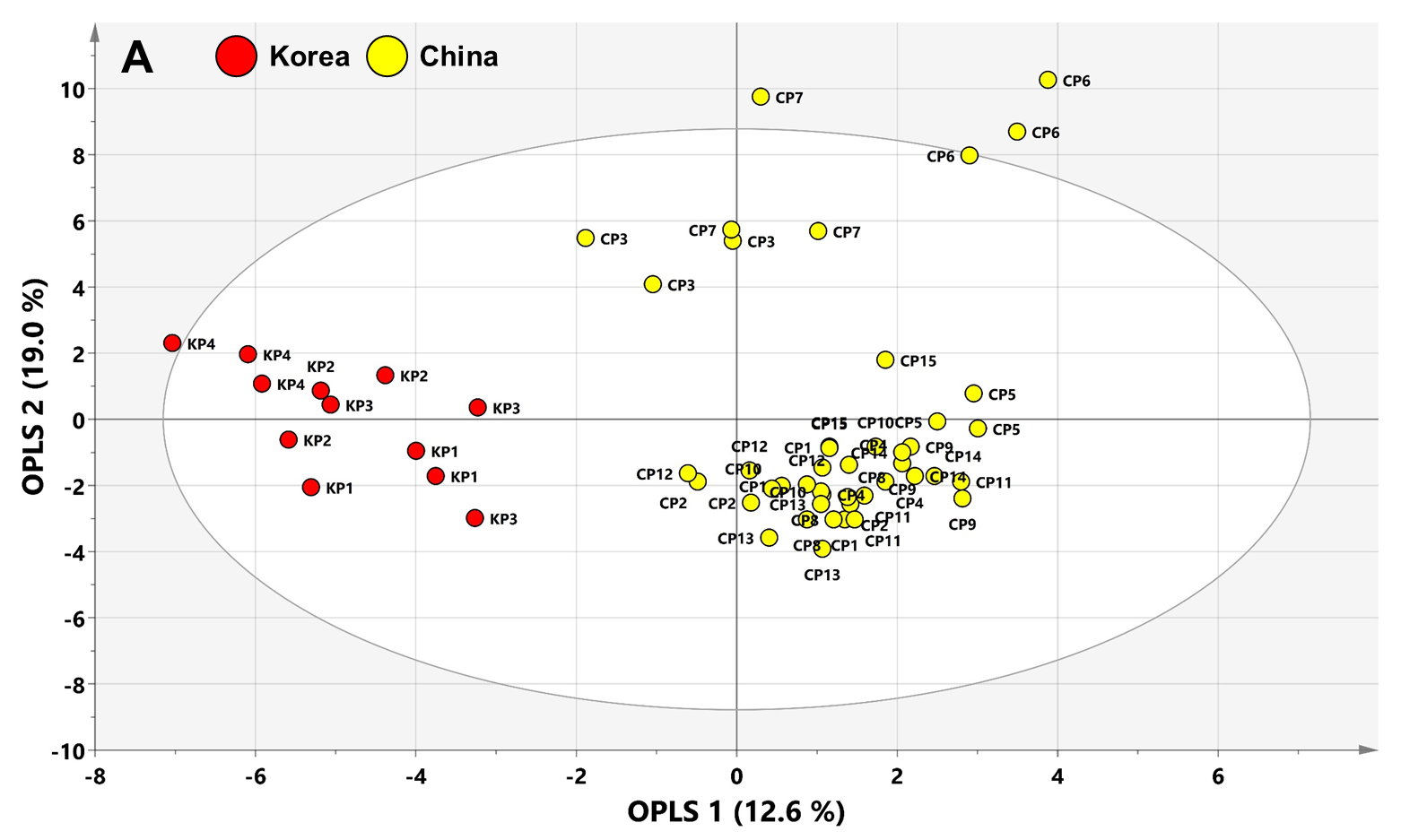


Figure S3. OPLS-DA score (A) and loading (B) plots of perilla (*Perilla frutescens*) seeds from Korea and China.
C20-ol, eicosanol; C21-ol, heneicosanol; C22-ol, docosanol; C23-ol, tricosanol; C24-ol, tetracosanol; C26-ol, hexacosanol; C27-ol, heptacosanol; C28-ol, octacosanol; C30-ol, triacontanol; C12:0, lauric acid; C14:0, myristic acid; C16:1n7, palmitoleic acid; C16:0, palmitic acid; C18:2n6, linoleic acid; C18:3n3, α-linolenic acid; C18:1n9, oleic acid; C18:0, stearic acid; C20:0, arachidic acid; C22:0, behenic acid; C24:0, lignoceric acid.

(*Continued next page*)

*(Figure S3., continued)*


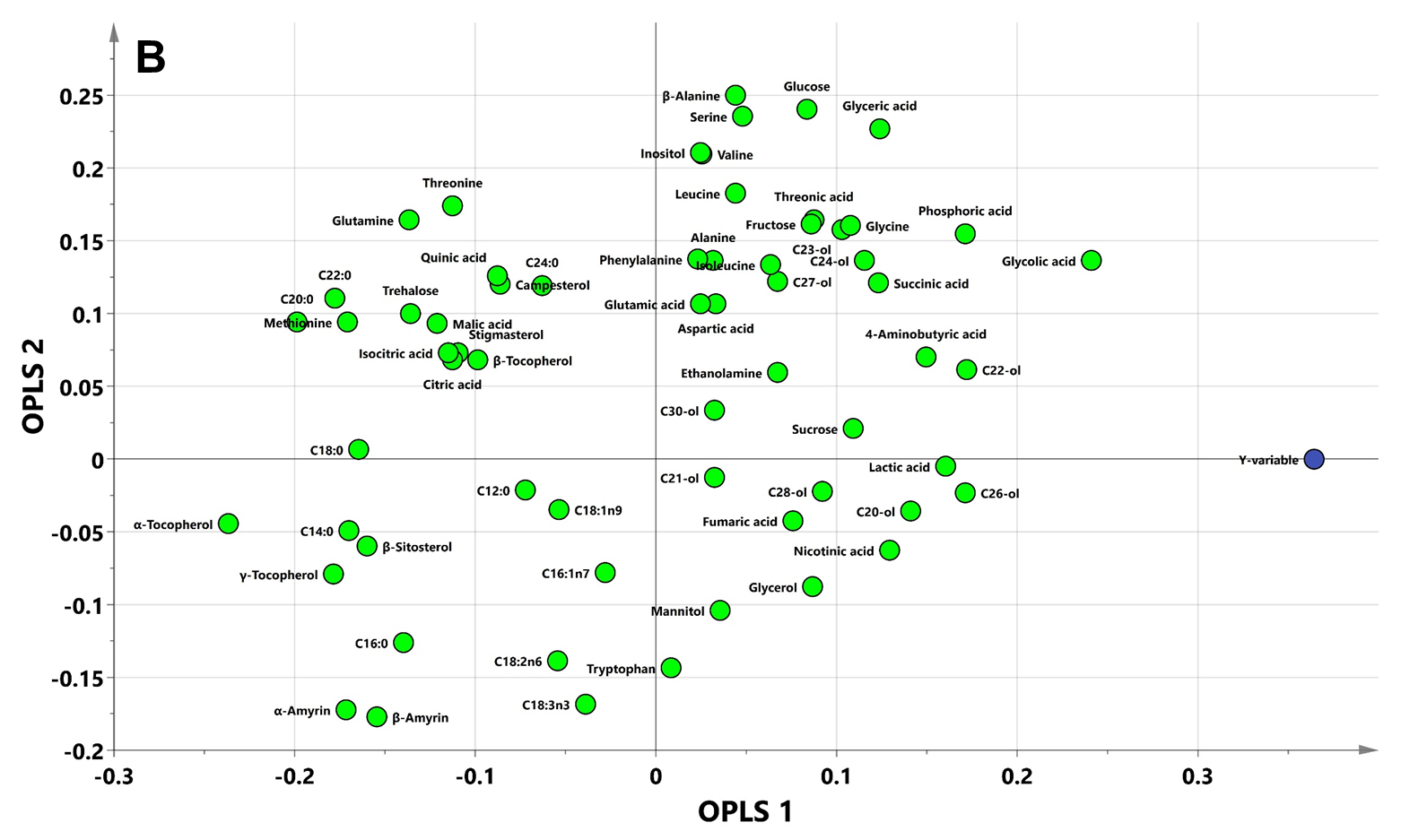


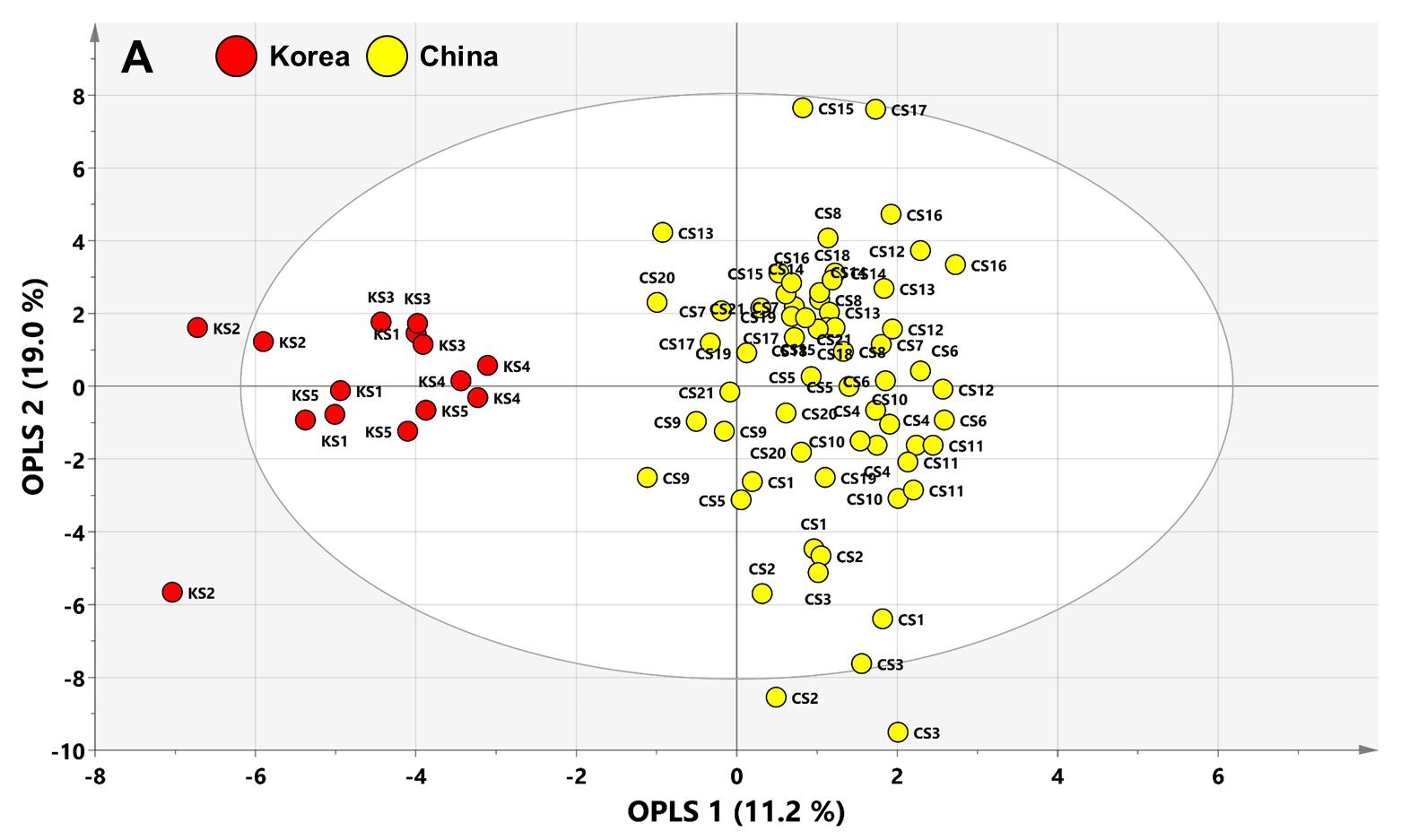


Figure S4. OPLS-DA score (A) and loading (B) plots of sesame (*Sesamum indicum*) seeds from Korea and China.
C20-ol, eicosanol; C21-ol, heneicosanol; C22-ol, docosanol; C23-ol, tricosanol; C24-ol, tetracosanol; C26-ol, hexacosanol; C27-ol, heptacosanol; C28-ol, octacosanol; C30-ol, triacontanol; C12:0, lauric acid; C14:0, myristic acid; C16:1n7, palmitoleic acid; C16:0, palmitic acid; C18:2n6, linoleic acid; C18:3n3, α-linolenic acid; C18:1n9, oleic acid; C18:0, stearic acid; C20:0, arachidic acid; C22:0, behenic acid; C24:0, lignoceric acid.

(*Continued next page*)


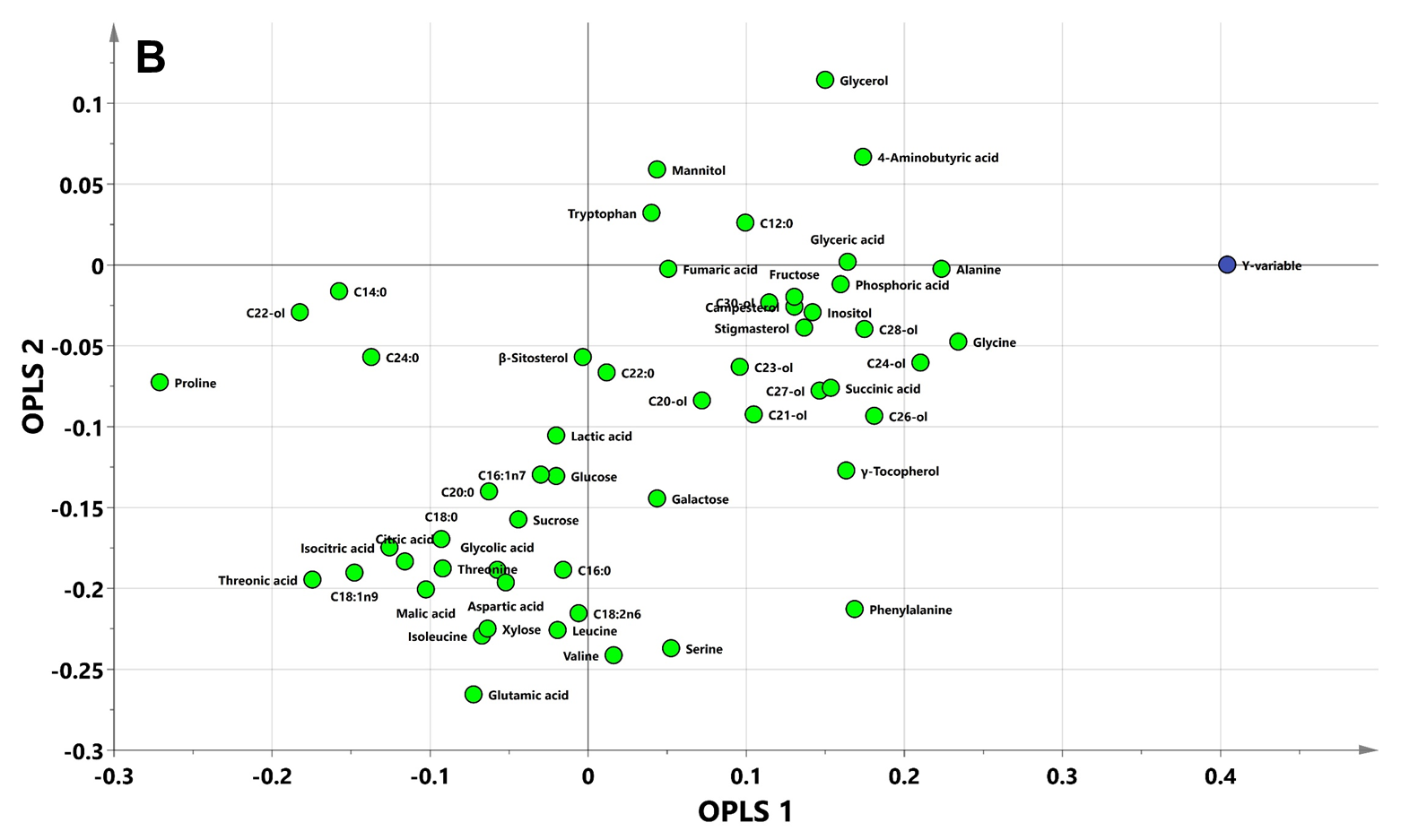
*(Figure S4., continued)*


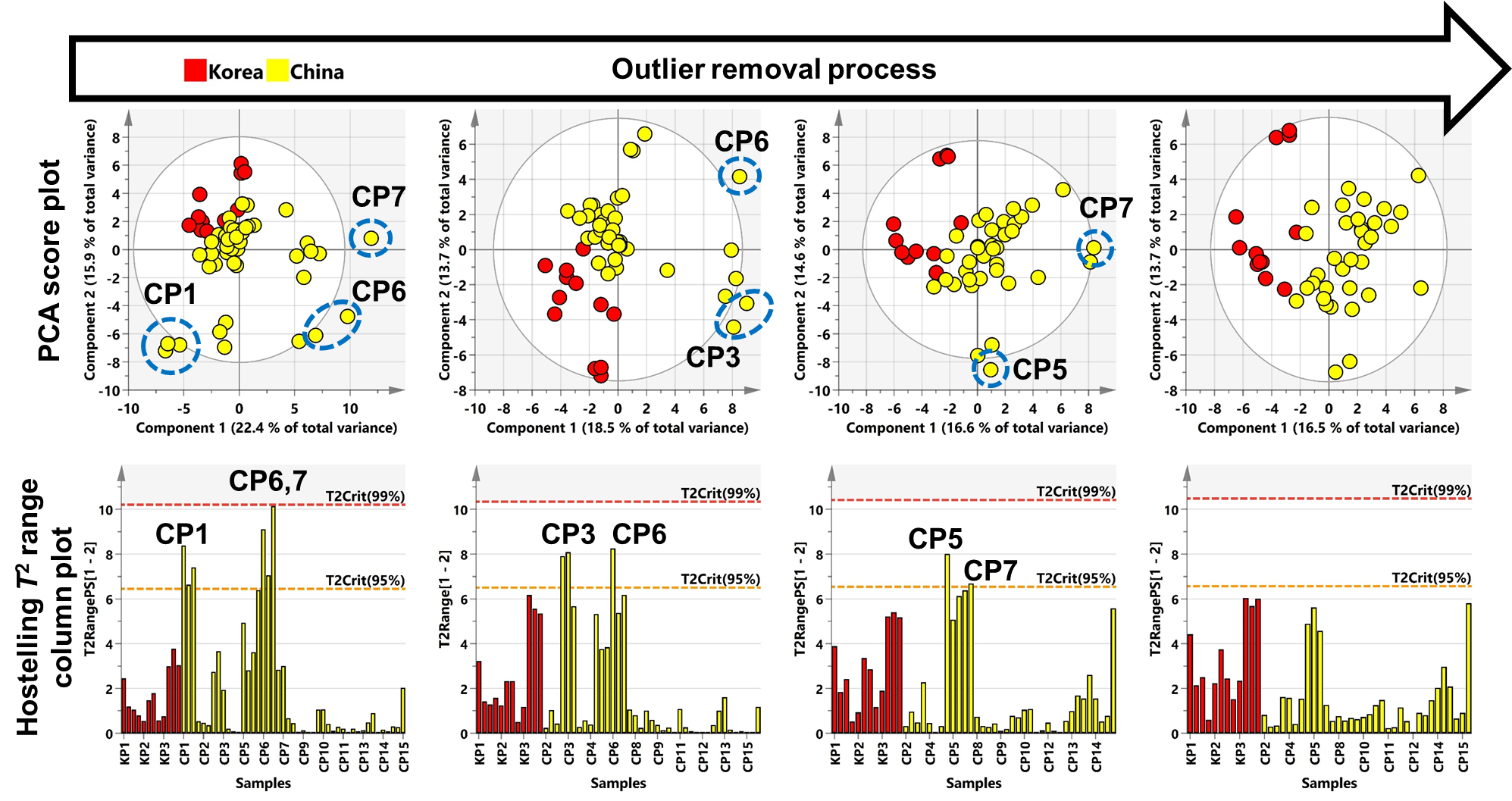
 Figure S5. PCA score plots and Hostelling *T*^2^ range column plots of perilla (*Perilla frutescens*) seeds from Korea and China for outlier removal process.
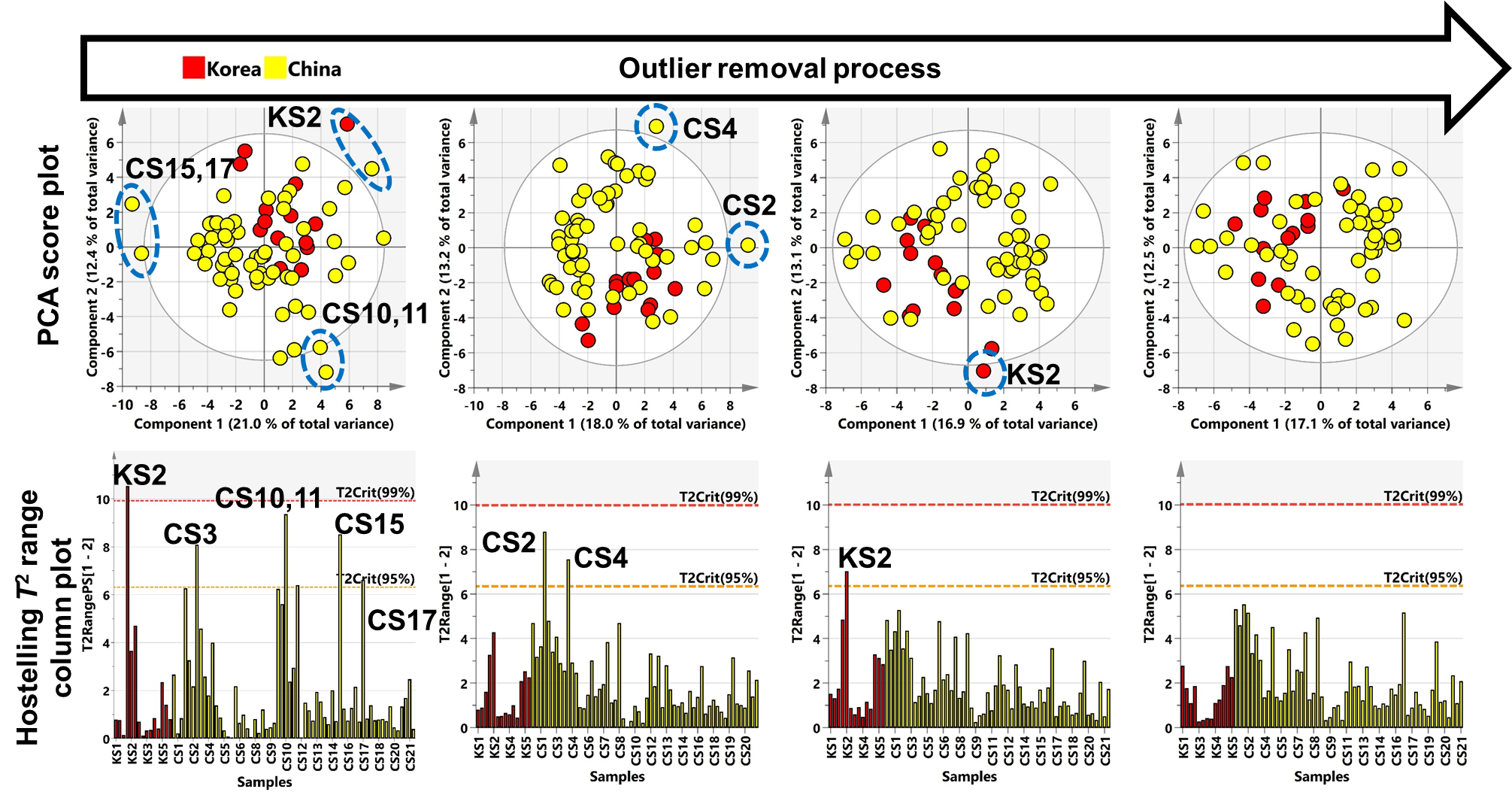
 Figure S6. PCA score plots and Hostelling *T*^2^ range column plots of sesame (*Sesamum indicum*) seeds from Korea and China for outlier removal process.


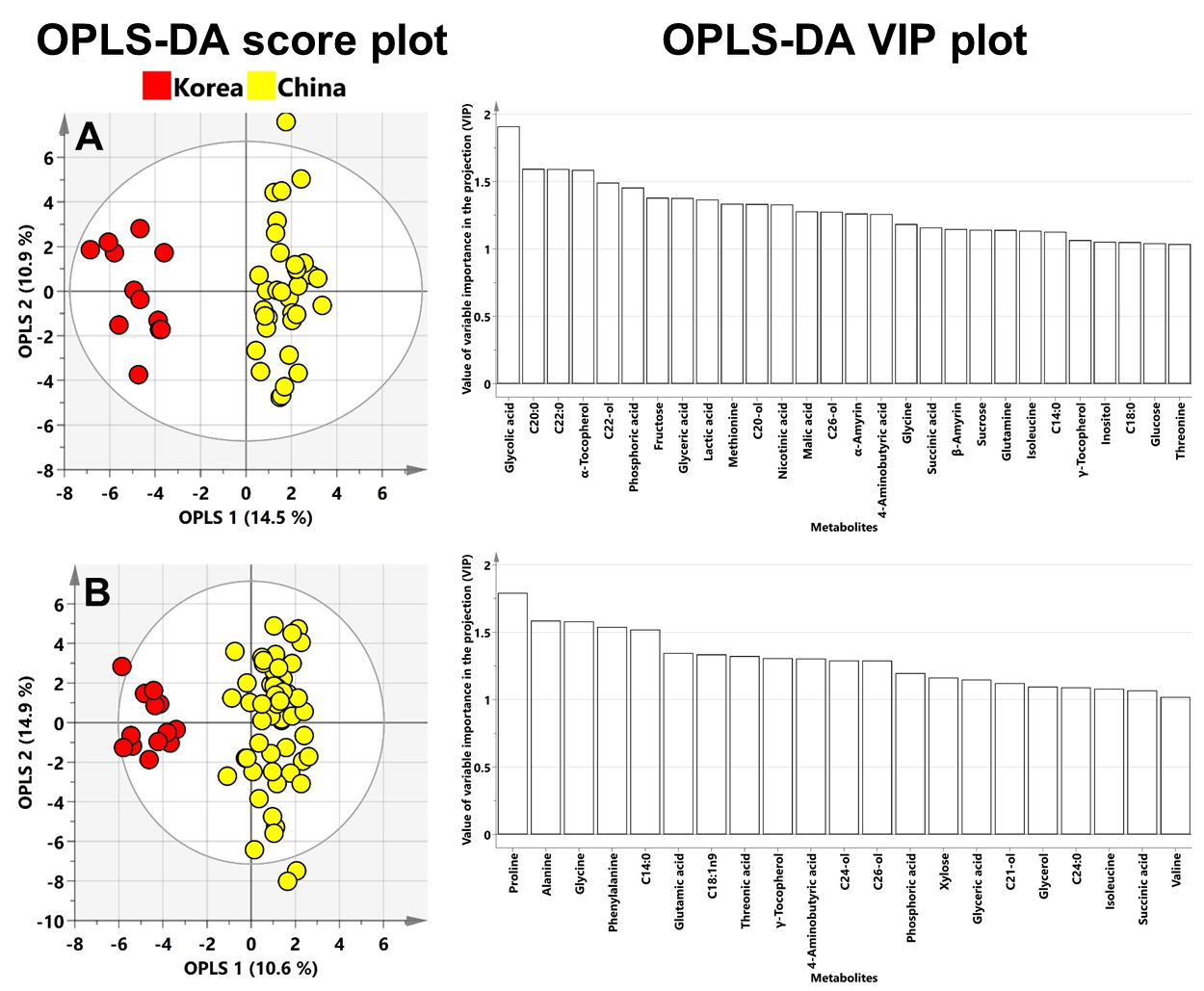
Figure S7. OPLS–DA score plots and VIP (variable importance in the projection) plots of perilla (A) and sesame (B) seeds from Korea and China with outliers removed dataset. C20-ol, eicosanol; C21-ol, heneicosanol; C22-ol, docosanol; C23-ol, tricosanol; C24-ol, tetracosanol; C26-ol, hexacosanol; C27-ol, heptacosanol; C28-ol, octacosanol; C30-ol, triacontanol; C12:0, lauric acid; C14:0, myristic acid; C16:1n7, palmitoleic acid; C16:0, palmitic acid; C18:2n6, linoleic acid; C18:3n3, α-linolenic acid; C18:1n9, oleic acid; C18:0, stearic acid; C20:0, arachidic acid; C22:0, behenic acid; C24:0, lignoceric acid.
